# Exon junction complexes regulate osteoclast‐induced bone resorption by influencing the NFATc1 m6A distribution through the “shield effect”

**DOI:** 10.1002/ctm2.70266

**Published:** 2025-03-06

**Authors:** Bao Sun, Jin‐Gang Yang, Zhe Wang, Zheng Wang, Wei Feng, Xing Li, Sheng‐Nan Liu, Jiang Li, Ya‐Qin Zhu, Ping Zhang, Wei Wang

**Affiliations:** ^1^ Department of Oral Pathology Shanghai Ninth People's Hospital, Shanghai Jiao Tong University School of Medicine; College of Stomatology, Shanghai Jiao Tong University; National Center for Stomatology; National Clinical Research Center for Oral Diseases; Shanghai Key Laboratory of Stomatology Shanghai China; ^2^ Department of Stomatology Tongren Hospital, Shanghai Jiao Tong University School of Medicine Shanghai China; ^3^ Department of Gynecology First Hospital of Shanxi Medical University Taiyuan China; ^4^ Concordia Institute for Information Systems Engineering, Concordia University Montreal QC Canada; ^5^ Department of Endodontics Central Laboratory of Jinan Stomatological Hospital, Jinan Key Laboratory of Oral Tissue Regeneration, Shandong Provincial Health Commission Key Laboratory of Oral Diseases and Tissue Regeneration Jinan Shandong People's Republic of China; ^6^ Department of Oral Maxillofacial‐Head Neck Oncology Shanghai Ninth People's Hospital, Shanghai Jiao Tong University School of Medicine; College of Stomatology, Shanghai Jiao Tong University; National Center for Stomatology; National Clinical Research Center for Oral Diseases; Shanghai Key Laboratory of Stomatology Shanghai China; ^7^ Department of General Dentistry Shanghai Ninth People's Hospital, Shanghai Jiao Tong University School of Medicine; College of Stomatology, Shanghai Jiao Tong University; National Center for Stomatology; National Clinical Research Center for Oral Diseases; Shanghai Key Laboratory of Stomatology Shanghai China; ^8^ Department of Oral and Maxillofacial Surgery Affiliated Hospital of Stomatology, Nanjing Medical University Nanjing China

**Keywords:** exon junction complexes, m6A distribution characteristics, osteoclast, osteoporosis, shield effect

## Abstract

**Background:**

The distribution of the m6A methylation modification on the transcriptome is highly regionally selective and is mainly concentrated in abnormally long exons and stop codons. However, in‐depth research on the selective mechanism of m6A methylation is still lacking.

**Methods:**

In this research, meRIP sequencing, mRNA sequencing, meRIP, luciferase reporter assays and CRISPR/Cas9 conditional knockout mice were used to elucidate the distribution characteristics of NFATc1 m6A.

**Results:**

METTL14 controls osteoclast‐mediated bone resorption by means of the methylation (4249 A) of the NFATc1 gene during osteoclast differentiation. Exon junction complexes (EJCs) selectively protect the m6A methylation sites of the NFATc1 gene. When the methylation sites are located within short exon fragments (50–200 nt), EJCs prevent their hypermethylation and degradation through the “shield effect”; when the methylation sites are located in the 3′ UTR region or long exon fragments (greater than 300 nt), the “shield effect” disappears. Downstream, YTHDF2 induced the degradation of hypermethylation NFATc1 transcripts without site restriction.

**Conclusions:**

EJCs act as “shields” to regulate the m6A region selectivity of the NFATc1 gene, thereby determining the characteristics of m6A distribution in the gene. Importantly, EJCs can raise the level of m6A methylation of NFATc1 and degrade its mRNA, thereby inhibiting osteoclast differentiation and preserving bone mass. These results will be helpful for identifying potential molecular targets for osteoporosis treatment.

**Key points:**

- METTL14 controls osteoclast‐mediated bone resorption by means of the methylation (4249 A) of the NFATc1 gene during osteoclast differentiation.
- Exon junction complexes (EJCs) protect the remaining methylation sites of the NFATc1 gene (located in the inner exon fragment of 50–200 nt) from hypermethylation and degradation.
- The “shield effect” disappears when the exon fragment is extended to 300 nt. Downstream, YTHDF2 induced the degradation of hypermethylation NFATc1 transcripts without site restriction.

## Graphical abstract

EJCs protected the methylation sites of the NFATc1 gene (located in the inner exon fragment of 50–200 nt) from hypermethylation and degradation, and the “shields” disappeared when the exon fragment was extended to 300 nt. Downstream, YTHDF2 induced the degradation of NFATc1 transcripts without site restriction. EJCs act as “shields” to regulate the m6A region selectivity of the NFATc1 gene, thereby determining the characteristics of the m6A distribution in this gene.

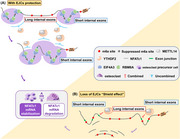

## 1 BACKGROUND

Decreased bone mass and the destruction of the microstructure of the bone are the hallmarks of osteoporosis, a common systemic bone disease that increases the risk of bone fracture.^1^, ^2^, ^3^, ^4^ As the population ages, the incidence of osteoporosis increases annually.^5^ Bone is a dynamically balanced organ that is constantly rebuilt throughout a person's life.^6^ A variety of pathogenic factors of abnormal bone metabolism can disrupt the network of bone homeostasis, exceed the compensatory effects of regulatory factors in vivo, and lead to excessive bone resorption or abnormal bone deposition.^7^, ^8^, ^9^ Bidirectional regulation of osteogenesis and osteolysis occurs in bone metabolic diseases.^10^, ^11^ However, in the field of bone metabolism, especially in osteoclasts, few studies on RNA methylation have been conducted. Our previous studies revealed that m6A methylation has a crucial function in the pathogenesis of osteoclast‐induced bone resorption^12^, ^13^

N6‐methyladenosine (m6A) has a crucial function in various physiological and pathophysiological mechanisms.^14^, ^15^, ^16^ The METTL3‐METTL14 methyltransferase complex recognizes a common DRACH sequence motif on mRNA; however, only around 5% of DRACH sequences of biological transcripts are chosen for methylation.^17^ Furthermore, the transcriptome distribution of m6A shows a clear regional selectivity, with substantial abundance in abnormally long internal exons and around stop codons.^18^, ^19^ The molecular mechanisms underlying m6A specificity remain poorly understood despite the critical role that selective m6A deposition plays in m6A‐mediated gene regulation. In 2023, He Chuan's team discovered that exon junction complexes (EJCs) play a crucial part in the abovementioned process. These structures function as m6A suppressors, preventing the methylation of exon junction proximal RNA in coding regions and controlling mRNA stability. Multiple features of mRNA m6A specificity are caused by the suppression of m6A by EJCs, and the local range of EJC protection is adequate to prevent m6A deposition in average length internal exons but not in terminal or long internal exons.^20^ EJCs have a variety of functions in the regulation of gene expression and are placed by spliceosomes in mRNAs 24 nt upstream of exon junctions.^21^, ^22^, ^23^, ^24^ This study has important significance for the illustration of the specificity of the m6A methylation distribution in the transcriptome and the mechanism of local mRNA packaging regulated by the exon structure. What mechanism leads to this splice‐dependent and exon length‐regulated inhibition of m6A methylation? Do EJCs affect the distribution of m6A methylation in bone tissue?

Based on these findings, we speculated that EJCs might act as m6A “shields” to regulate osteoclast differentiation. Therefore, RNA sequencing and m6A immunoprecipitation (meRIP) sequencing were conducted after RANKL‐induced osteoclast differentiation. The target genes that intersected with the two groups were chosen. The nuclear factor of activated T cells type C1 (NFATc1) gene, which has a high correlation with osteoclast differentiation, was selected for further study. We investigated the connection between the NFATc1 gene's m6A methylation level and osteoclast‐induced bone resorption by verifying functional m6A methylation sites in this gene. We next examined the posttranscriptional regulation of the NFATc1 gene by two important EJC factors, EIF4A3 and RBM8A.^25^ M6A specificity is controlled by “shields” that avoid m6A deposition in unmethylated transcriptomic regions of the NFATc1 mRNA. We identified EJC factors (EIF4A3 and RBM8A) as m6A “shields” that prevent exon junction proximal RNA within coding sequences from methylation and modulate the NFATc1 in osteoclasts through m6A suppression.

## 2 MATERIALS AND METHODS

### 2.1 Antibodies, cell line, and human subjects

The Shanghai Cell Center provided the murine RAW264.7 monocytic cell line. We acquired mouse RANKL from R&D Systems. Cell Signaling Technology provided specific antibodies against β‐actin (#4970), NFATc1 (#8032), METTL14 (#51104) and YTHDF2 (#71283). Abcam provided specific antibodies against horseradish peroxidase‐conjugated goat anti‐rabbit (ab205718), goat anti‐rabbit (Alexa Fluor) (ab150077), m6A (ab284130), METTL3 (ab195352), Ctsk (ab19027), MMP9 (ab38898), GAPDH (ab181602), and EIF4A3 (ab32485). Solarbio provided a specific antibody against RBM8A (K109688P).

Alveolar bone specimens were obtained from female donors aged 65–75 years who had undergone dental extraction at least one year prior and were scheduled for dental implantation at the affiliated stomatology hospital of Nanjing Medical University. During the implantation procedure, alveolar bone was harvested using a ring drill from postmenopausal (65–75 years old) women, distinguishing between those diagnosed with osteoporosis and those without.

### 2.2 Osteoclast differentiation, F‐actin band staining, and tartrate‐resistant acid phosphatase staining

RAW264.7 cells were seeded and maintained in DMEM enriched with 10% fetal bovine serum and 1% penicillin/streptomycin antibiotic mix. Experimental treatments were administered according to the study's protocol. Subsequently, cells were stimulated with RANKL at a concentration of 20 ng/mL for a period of 7 days. The culture medium was replenished every second day to ensure optimal conditions. After the 7‐day stimulation period, the extent of osteoclast differentiation was quantified through tartrate‐resistant acid phosphatase (TRAP) staining. The cells were fixed and stained for TRAP activity following the methodology detailed in our earlier research.^13^ Osteoclasts were identified as multinucleated TRAP‐positive cells harboring a minimum of three nuclei. In parallel, RAW264.7 cells were seeded and cultured under identical conditions for 7 days. Following fixation, the cells underwent staining for F‐actin band. Briefly, cells were permeabilized with .5% Triton X‐100 for 5 min, allowing for subsequent staining with 5 µL/mL phalloidin (Yeasen) for 30 min.

### 2.3 Western blot analysis

Total proteins were extracted from cultured cells using RIPA lysis buffer. Equal amounts of proteins were separated by electrophoresis on a SurePAGE gel (GenScript) and then transferred onto polyvinylidene fluoride (PVDF) membranes (Millipore). The PVDF membranes were incubated overnight at 4°C with primary antibodies. The membranes were incubated with secondary antibodies after washing. Enhanced chemiluminescence (ECL) reagents (Millipore) were used to visualize the protein bands.

### 2.4 RT‐qPCR analysis

Total RNA was isolated from samples using the TRIzol reagent, followed by reverse transcription utilizing the PrimeScript RT Master Mix RR036A. Custom‐designed primers, detailed in Table S1, were synthesized by Sangon Biotech for the amplification of target transcripts. RT‐qPCR was executed with the SYBR Premix Ex Taq RR420A, enabling the quantification of gene expression. The relative expression levels of the genes of interest were determined using the 2^−∆∆Ct^ method. GAPDH served as normalization, and the data were compared to normalized values of the control. Furthermore, the specific siRNA sequences employed in knockdown experiments are catalogued in Table S2.

### 2.5 M6A immunoprecipitation

The meRIP assay was performed with the Magna MeRIP m6A kit (Millipore) to assess the m6A modifications of individual transcripts. Initially, total RNA was extracted from cells that had undergone pre‐treatment. Subsequently, the RNA was processed to yield fragments of approximately 100 nucleotides in length, facilitating efficient immunoprecipitation. The fragmented RNA samples were subjected to immunoprecipitation using magnetic beads that had been precoated with an antibody specific to m6A or anti‐IgG for background subtraction. The m6A modified RNA fragments were selectively eluted using N6‐methyladenosine 5′‐monophosphate sodium salt. Post‐elution, the enriched RNA fragments underwent RT‐qPCR analysis. The primers for RT‐qPCR were custom‐designed based on the guidance provided by the SRAMP website (http://www.cuilab.cn/sramp/). They are listed in Table S3. The input was used to normalize the relative enrichment of m6A.

### 2.6 Dot blot and m6A quantification

Post‐heating, RNA samples were immobilized onto a positively charged nylon membrane (Beyotime). The presence of m6A modifications was probed using a specific m6A antibody, and detection was facilitated by ECL reagent (Millipore). As a control, the same membrane was also stained with methylene blue. For immunofluorescence staining, fixed cells were incubated with an m6A antibody and subsequently exposed to a fluorescent dye‐labeled secondary antibody. The outcomes were meticulously examined using a confocal laser scanning microscope (Leica Microsystems).

### 2.7 Luciferase reporter assay

Cells were transfected with a pmirGLO‐based luciferase vector, incorporating distinct mutated segments of the NFATc1 gene. Si‐EIF4A3/RBM8A or si‐NC was cotransfected. The Dual Luciferase Reporter Assay System (Promega) was utilized to quantitatively determine both renilla and firefly luciferase activities. Renilla luciferase activity was applied to normalize firefly luciferase activity to evaluate the translational efficiency of the reporter constructs. The experiments were executed in triplicate.

### 2.8 RNA immunoprecipitation

Adhering to the manufacturer's instructions, the RNA immunoprecipitation (RIP) assay was conducted utilizing the Magna RIP Kit (Millipore). Essentially, magnetic beads were preincubated with either anti‐YTHDF2 or anti‐IgG antibodies before being combined with the cell lysates. After isolating the antibody‐bound protein complexes, the RNAs of interest were selectively eluted, purified, and subjected to RT‐qPCR analysis for quantitative assessment. The degree of relative enrichment was normalized to the input control.

### 2.9 Fluorescence in situ hybridization

A probe targeting NFATc1 was used for RNA fluorescence in situ hybridization (FISH). After hybridization, the RAW264.7 cells were labeled with an anti‐digoxin antibody. The following day, the cells were stained with goat anti‐rabbit IgG (shown in red). DAPI was used to label nuclear DNA (shown in blue). We detected the YTHDF2 protein in RAW264.7 cells via an immunofluorescence assay. An anti‐YTHDF2 antibody and a goat anti‐rabbit antibody were used to detect the YTHDF2 protein (shown in green). These images were captured applying a fluorescence microscope. The probes used for FISH are listed in Table S4.

### 2.10 Generation of conditional EIF4A3/RBM8A knockout mice

For conditional knockout, EIF4A3^fl/+^ mice on a C57BL6/J background were generated utilizing the CRISPR‐Cas9 gene editing system. Specifically, exon 2 of EIF4A3 was the target of interest in the generation of the conditional knockout of the floxed EIF4A3 allele. Using the CRISPR design tool (http://crispr.tefor.net), sgRNAs were designed to target regions adjacent to exon 2. Target efficacy was evaluated using a standard CRISPR activity assay (GenScript). A donor vector contained with Lox P sites flanking exon 2 was mixed with Cas9 mRNA and sgRNAs and co‐injected into the cytoplasm of one‐cell‐stage C57BL6/J zygotes. The injected zygotes were then implanted into the oviducts of Kunming pseudo‐pregnant females to generate F0 mice. Genotyping of F0 progeny was conducted via PCR and sequencing of tail genomic DNA to identify individuals harboring the desired modifications. Subsequently, these mice were crossed with wild‐type C57BL6/J mice to establish germline transmission in the F1 generation. Both PCR genotyping and DNA sequencing were employed to confirm the accuracy of the EIF4A3^fl/+^ genotype. Ctsk‐Cre transgenic mice were sourced from Shanghai Model Organisms. We crossed male Ctsk‐Cre mice with EIF4A3^fl/+^ mice to generate Ctsk‐Cre; EIF4A3^fl/+^ mice and heterozygous conditional EIF4A3 knockout mice. By mating Ctsk‐Cre; EIF4A3^fl/+^ mice with EIF4A3^fl/+^ female mice, we generated Ctsk‐Cre; EIF4A3^fl/fl^ mice as homozygous conditional EIF4A3 knockout mice. An analogous breeding strategy was implemented to generate conditional RBM8A knockout mice, with exons 2–4 of RBM8A serving as critical targets. The genotyping of these transgenic mice was achieved through PCR analysis of genomic DNA extracted from tail samples. The primers applied for the detection of EIF4A3/RBM8A are listed in Table S5. All animals were bred and maintained under specific‐pathogen‐free conditions. Eight‐week‐old female conditional EIF4A3/RBM8A knockout mice and their littermate controls underwent either OVX or a sham operation. After anesthesia, both ovaries were surgically removed. Upon locating the ovaries, we exposed the fallopian tubes, ligated them, and removed the ovaries. In contrast, the sham surgery group underwent ovarian exposure without removal, returning the ovaries to the abdominal cavity before closing the incision. Post‐surgery, mice from both the sham and OVX groups were recuperated for an additional 8 weeks.

### 2.11 Micro‐CT, histological detection, and immunohistochemistry

Micro‐CT imaging of tibiae was performed using a SkyScan 1176 system (Bruker) at a scanning resolution of 9 µm, with settings of 50 kV and 450 µA. Trabecular bone analysis was focused on a region of interest along the long axis of the proximal tibia and 1–3 mm away from the growth plate. The bone histomorphometric parameters included trabecular number (Tb.N), trabecular bone volume/total volume (BV/TV), trabecular thickness (Tb.Th), trabecular bone mineral density (BMD) and trabecular separation (Tb.Sp). Serial sections were prepared for the subsequent histological analysis. Histological alterations were visualized using hematoxylin and eosin (H&E) staining. Immunohistochemistry was conducted by incubating the sections with primary antibodies directed against Ctsk, MMP9, or NFATc1. After washing, the sections were incubated with secondary antibodies. Immunoreactivity was visualized using diaminobenzidine (Sigma‐Aldrich). TRAP staining was performed according to established protocols.

### 2.12 Fluorescence labeling analysis

To observe the mineralizing front, mice received subcutaneous injections of 20 mg/kg calcein (Sigma) 8 days before euthanasia and, subsequently, 30 mg/kg alizarin red S (Sigma) 2 days prior. The undecalcified bone samples were processed through dehydration and embedded within pure resin blocks. They were cut along the center of the tibia axis to produce thin sections. Images were employed to visualize new bone formation. Histomorphometric analyses, encompassing measurements of mineralizing surface/bone surface (MS/BS), mineral apposition rate (MAR), and bone formation rate/bone surface (BFR/BS), were conducted to assess the bone dynamics.

### 2.13 Statistical analysis

Statistical analyses were executed utilizing SAS software, version 9.4 (SAS Institute). Normality tests were conducted to evaluate the normality of continuous data before appropriate methods of statistical description or analysis were applied. A parametric Student's *t*‐test was applied if the data were normally distributed. Instead, the nonparametric Wilcoxon rank sum test or Kruskal–Wallis (*H* test) was applied if the data did not meet the requirements for the parametric test. Statistical significance was established at a threshold of *p* < .05.

## 3 RESULTS

### 3.1 Differentially expressed genes and high‐throughput sequencing

**FIGURE 1:**
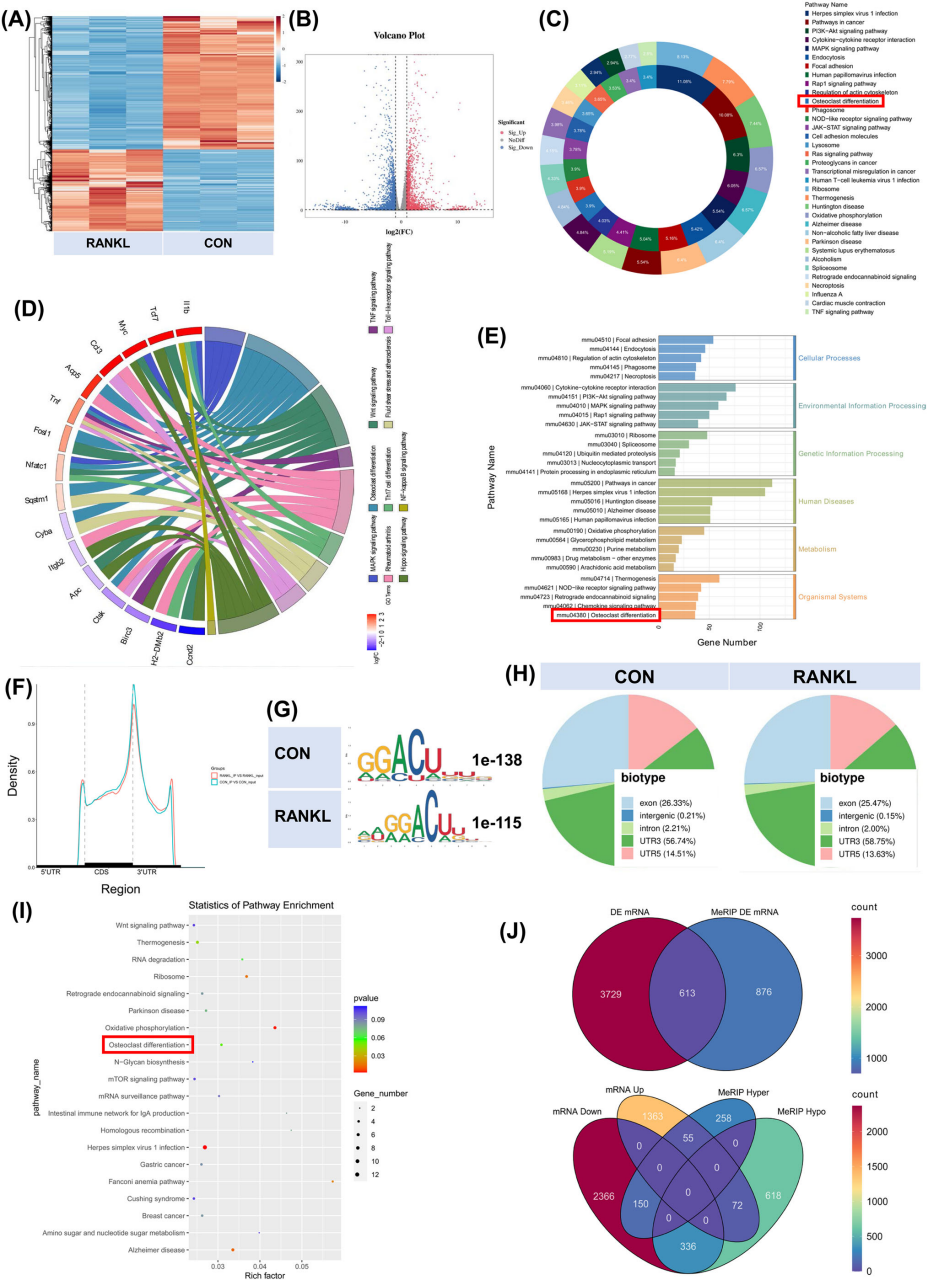
High‐throughput sequencing and differentially expressed genes. Collect RAW264.7 cells with or without RANKL stimulation. Total RNA was extracted from the two groups for RNA‐seq and m6A‐seq analysis. (A, B) Heatmap and volcano plot displaying differentially expressed mRNAs between the CON and RANKL groups. (C) The top 20 KEGG pathways enriched in upregulated and downregulated genes were selected to construct a Donut chart. The number marked on each figure is the proportion of the specified KEGG pathway in the top 20 pathways (pathways with a proportion below 2% are not marked) (osteoclast differentiation is boxed in red). (D) The KEGG terms are visualized in a chord plot. (E) The top five significant KEGG pathways were enriched in cellular processes, environmental information processing, genetic information processing, human diseases, metabolism and organismal systems. The horizontal coordinate is the number of differentially expressed genes contained in the pathway, and the different colors along the vertical coordinate represent the primary classification from the KEGG enrichment analysis (osteoclast differentiation is boxed in red). (F) Enrichment profiles of m6A in the mRNA transcriptome of the two groups. (G) Predominant consensus motifs found in the m6A‐seq peaks of both groups. (H) An m6A peak distribution plot showing the proportion of total m6A peaks in both groups. (I) A bubble chart showing the enrichment of the top 20 biological processes associated with the differentially expressed m6A methylated genes (osteoclast differentiation is shown in a red box). (J) A Venn diagram shows 613 intersecting genes with differential expression and differential m6A methylation in both groups (top panel). The differentially expressed genes are classified according to mRNA and m6A methylation levels (bottom panel).

We collected RAW264.7 cells, either inducing RANKL or not. Total RNAs were subjected to m6A‐seq and RNA‐seq. A volcano plot and heatmap were generated to show the differentially expressed mRNAs between the CON and RANKL groups (Figure 1A,B). Kyoto Encyclopedia of Genes and Genomes (KEGG) enrichment analysis of genes whose expression changed after RANKL stimulation showed the enrichment of osteoclast differentiation. Differentially expressed genes in osteoclast differentiation include Jun, Mapk3, Ncf1, Fos, Plcg2, Sirpa, Spi1, and Tgfbr2. The intersection genes of signaling pathways such as NF‐kappa B, TNF, macrophage differentiation, rheumatoid arthritis and Ras are also considered to be closely related to the osteoclast differentiation function (Figure 1C–E). These m6A modifications were enriched near stop codons in both groups (Figure 1F). We used independent biological replicates to map the m6A motif. The GGACU motif was significantly enriched within the m6A site (Figure 1G). Similar patterns of m6A distribution were noticed in both groups (Figure 1H). The enrichment of biological processes associated with the differentially expressed m6A‐methylation genes is displayed in a bubble chart. The top 20 biological functions are exhibited, and osteoclast differentiation is labelled in the red frame (Figure 1I). The *p*‐value for differentially expressed genes from m6A‐seq was less than .05, while the threshold for differentially expressed genes from RNA‐seq was set to more than 1.5 (Figure 1J).

### 3.2 RANKL lowers the m6A methylation level and promotes osteoclast differentiation in osteoclast precursor cells

**FIGURE 2:**
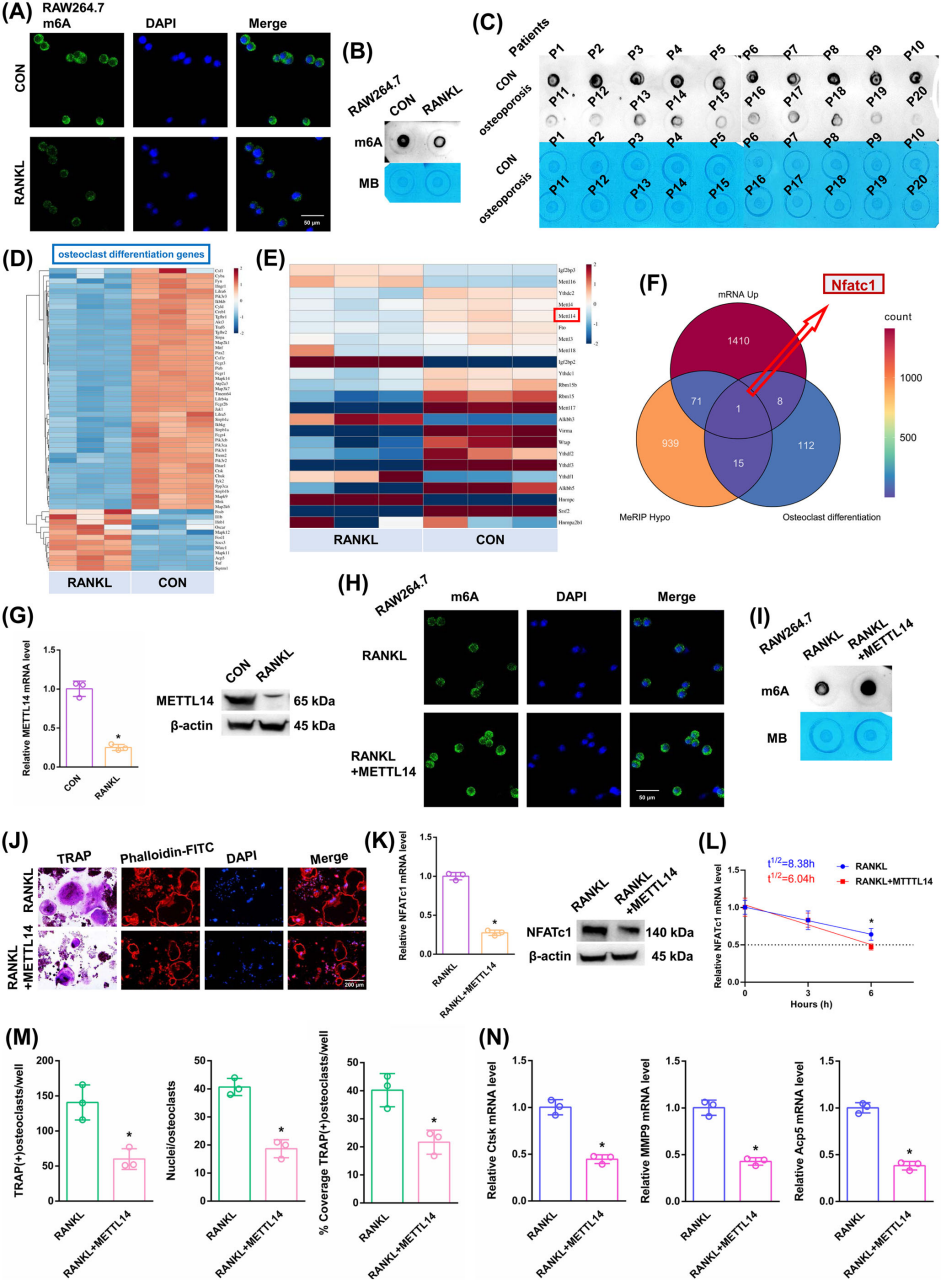
The effects of RANKL on the m6A methylation level in osteoclast precursor cells. (A, B) Immunofluorescence staining and m6A dot blot assays demonstrating the overall m6A levels in RAW264.7 cells after RANKL stimulation. Scale bar: 50 µm. (C) m6A dot blot analysis shows the overall m6A levels in samples from postmenopausal women with or without osteoporosis. (D) A heatmap displaying the differentially expressed genes associated with osteoclast differentiation after RANKL stimulation. (E) A heatmap illustrating the differentially expressed m6A methyltransferase‐related enzymes after RANKL stimulation. (F) KEGG analysis revealing the differentially expressed gene (NFATc1) identified from MeRIP down genes, mRNA hyper genes and osteoclast differentiation genes. (G) The mRNA and protein expression levels of METTL14 in RAW264.7 cells after RANKL stimulation. (H, I) Immunofluorescence staining and m6A dot blot analysis show the overall m6A levels in RAW264.7 cells after stimulation with RANKL+METTL14. Scale bar: 50 µm. (J) TRAP staining and F‐actin band staining were employed to assess osteoclast differentiation after stimulation with RANKL+METTL14. Scale bar: 200 µm. (K) The mRNA and protein expression levels of NFATc1 in RAW264.7 cells after stimulation with RANKL+METTL14. (L) The half‐life of NFATc1 mRNA was estimated by linear regression analysis after stimulation with RANKL+METTL14. (M) Histograms display the number, coverage rate and nuclei of TRAP‐positive osteoclasts after stimulation with RANKL+METTL14. (N) Relative expression levels of Ctsk, MMP9, and Acp5 in RAW264.7 cells after stimulation with RANKL+METTL14. These data represent three independent experiments and are presented as the means ± SDs (*p < .05).

After RANKL stimulation, the overall m6A methylation level was significantly reduced, as shown by dot blot and immunofluorescence assays (Figure 2A,B). The m6A level was shown to be higher in the alveolar bone of postmenopausal women without osteoporosis (P1–P10) but lower in the alveolar bone of postmenopausal women with osteoporosis (P11–P20), according to a dot blot assay (Figure 2C). The heatmap displayed differentially expressed genes associated with osteoclast differentiation (Figure 2D). The expression of m6A “writers”, “erasers”, and “readers” was assessed in both groups. Among these candidates, the RANKL‐stimulated cells exhibited a significant decrease in METTL14 expression, which may account for the decrease in m6A methylation levels (Figure 2E). The expression of METTL14 mRNA decreased in the alveolar bone of postmenopausal women with osteoporosis (P11–P20) according to RT‐qPCR analysis (Figure S1A). KEGG analysis showed one differentially expressed gene, NFATc1, from MeRIP down genes, mRNA hyper genes and osteoclast differentiation genes (Figure 2F). NFATc1 is situated on chromosome 18: 80, 802, 994‐80, 904, 912, and consists of 10 exons (Figure S2A, Table S6). The NFATc1 expression was substantially increased after RANKL stimulation (Figure S1B). After RANKL stimulation, the Ctsk, MMP9 and Acp5 mRNAs increased (Figure S1C). We conducted an RNA stability assay to detect the effect of m6A on NFATc1 mRNA metabolism. The half‐life of NFATc1 was extended after RANKL stimulation (Figure S1D). The METTL14 expression was substantially decreased after RANKL stimulation (Figure 2G). This result suggests that a decrease in the m6A methylation level may play a crucial part in RANKL‐induced osteoclastic differentiation. We designed a rescue experiment involving the RANKL and RANKL+METTL14 groups. The transgenic cell line was successfully constructed (Figure S1E). After RANKL+METTL14 stimulation, the overall m6A methylation level was significantly increased, as shown by dot blot and immunofluorescence assays (Figure 2H,I). After RANKL+METTL14 stimulation, a decreasing number of osteoclasts with shrunken TRAP‐positive cell bodies and F‐actin bands and fewer nuclei were identified based on the results of F‐actin band and TRAP staining (Figure 2J). The number of and number of nuclei in TRAP‐positive osteoclasts were considerably reduced after RANKL+METTL14 stimulation, as indicated by the histogram coverage rate (Figure 2M). After RANKL+METTL14 stimulation, the Ctsk, MMP9 and Acp5 mRNAs decreased (Figure 2N). The NFATc1 expression was substantially decreased after RANKL+METTL14 stimulation (Figure 2K). The half‐life of NFATc1 was shortened after RANKL+METTL14 stimulation (Figure 2L). These findings showed that RANKL promoted osteoclast differentiation by reducing METTL14 expression to a certain extent.

### 3.3 The NFATc1 gene participates in the process by which RANKL promotes osteoclast differentiation by reducing METTL14 levels

**FIGURE 3:**
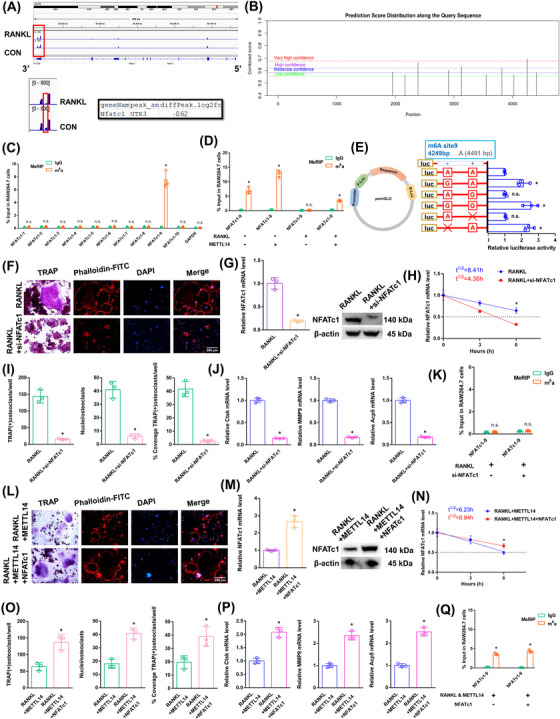
The effects of the NFATc1‐9 segment on the process of osteoclast differentiation. (A) A schematic diagram showing the genomic location of NFATc1. Visualization of m6A peaks within the NFATc1 transcript through meRIP‐Seq after RANKL stimulation, with m6A peaks located in the 3′ UTR of NFATc1. (B) Potential m6A methylation sites within NFATc1 based on the SRAMP website. (C) Enrichment of ten NFATc1 segments between the anti‐m6A group and the anti‐IgG group detected by m6A‐RT‐qPCR after RANKL stimulation. (D) Enrichment of NFATc1‐9 fragment detected by m6A‐RT‐qPCR after RANKL or METTL14 stimulation. (E) Validation of m6A modification sites through step‐by‐step mutation using luciferase reporters. (F) Assessment of osteoclast differentiation after RANKL+si‐NFATc1 stimulation through TRAP staining and F‐actin band staining. Scale bar: 200 µm. (G) Detection of NFATc1 mRNA and protein expression levels in RAW264.7 cells after RANKL+si‐NFATc1 stimulation. (H) Estimation of the half‐life of NFATc1 mRNA after RANKL+si‐NFATc1 stimulation through linear regression analysis. (I) Histograms displaying the number, coverage rate and nuclei of TRAP‐positive osteoclasts after RANKL+si‐NFATc1 stimulation. (J) Relative expression levels of Ctsk, MMP9, and Acp5 in RAW264.7 cells after RANKL+si‐NFATc1 stimulation. (K) Enrichment of NFATc1‐9 segment after RANKL or si‐NFATc1 stimulation detected by m6A‐RT‐qPCR. (L) Assessment of osteoclast differentiation after RANKL+METTL14+NFATc1 stimulation through TRAP staining and F‐actin band staining. Scale bar: 200 µm. (M) Detection of NFATc1 mRNA and protein expression levels in RAW264.7 cells after RANKL+METTL14+NFATc1 stimulation. (N) Estimation of the half‐life of NFATc1 mRNA after RANKL+METTL14+NFATc1 stimulation through linear regression analysis. (O) Histograms displaying the number, coverage rate and nuclei of TRAP‐positive osteoclasts after RANKL+METTL14+NFATc1 stimulation. (P) Relative expression levels of Ctsk, MMP9, and Acp5 in RAW264.7 cells after RANKL+METTL14+NFATc1 stimulation. (Q) Detection of enrichment of NFATc1‐9 segment after RANKL+METTL14 or NFATc1 stimulation by m6A‐RT‐qPCR. These data represent three independent experiments and are presented as the means ± SDs (*p < .05).

The NFATc1 m6A methylation level in the CDS‐3′ untranslated region (3′ UTR) of the mRNA in the RANKL group was notably lower than that in the CON group (Figure 3A; Figure S2B). The SRAMP website was applied to predict the abundance of m6A methylation loci in NFATc1. In total, 10 potential loci along NFATc1's length were identified (Figure 3B; Figure S2C). To identify the effective m6A‐methylated sequences in NFATc1, we designed ten pairs of primers (Figure S2D). The NFATc1 mRNA increased in the alveolar bone of postmenopausal women with osteoporosis (P11–P20) according to RT‐qPCR analysis (Figure S1F). Only the NFATc1‐9 segment showed a high level of m6A methylation in the CON group according to the m6A‐RT‐qPCR results (Figure 3C). The previously high level of the m6A methylated NFATc1‐9 segment dropped after RANKL stimulation. RANKL showed the same effect on reducing the NFATc1‐9 segment's m6A methylation level after METTL14 stimulation (Figure 3D). Next, the m6A modification site in the NFATc1‐9 segment was confirmed via step‐by‐step mutation of reporter plasmids. In particular, different mutant NFATc1 fragments were inserted into the luciferase reporters individually: 4249A‐G, 4249A‐Delete, 4491A‐G, and 4491A‐Delete. The NFATc1‐9 (4249 A) is an effective m6A methylation site (Figure 3E). We designed a rescue experiment involving the RANKL and RANKL+si‐NFATc1 groups to investigate the role of NFATc1 in RANKL‐induced osteoclast differentiation. The transgenic cell line with si‐NFATc1 was successfully constructed (Figure S1G). As demonstrated by the results of the aforementioned experiments, when we transfected si‐NFATc1, the downregulation of NFATc1 reduced osteoclast differentiation after RANKL stimulation (Figure 3F,I,J). The NFATc1 expression was substantially decreased after RANKL+si‐NFATc1 stimulation (Figure 3G). The half‐life of NFATc1 was remarkably shortened after RANKL+si‐NFATc1 stimulation in a rescue experiment (Figure 3H). The NFATc1‐9 segment showed a low m6A methylation level in both the RANKL and RANKL+si‐NFATc1 groups according to the m6A‐RT‐qPCR results (Figure 3K). These findings showed that the NFATc1 gene has a decisive role in RANKL‐induced osteoclast differentiation. We generated RANKL+METTL14 and RANKL+METTL14+NFATc1 groups to investigate the role of NFATc1 in RANKL‐induced osteoclast differentiation after METTL14 overexpression. The transgenic cell line was successfully constructed (Figure S1H). As demonstrated by the results of the aforementioned experiments, when we transfected NFATc1, the upregulation of NFATc1 increased osteoclast differentiation after RANKL+METTL14 stimulation (Figure 3L,O,P). The NFATc1 expression was substantially increased in the RANKL+METTL14+NFATc1 group (Figure 3M). The half‐life of NFATc1 was extended after RANKL+METTL14+NFATc1 stimulation (Figure 3N). The NFATc1‐9 segment showed the same level of m6A methylation in both groups according to the m6A‐RT‐qPCR results (Figure 3Q). These results proved that RANKL increased NFATc1 gene expression by reducing METTL14 expression during osteoclast differentiation. Specifically, the posttranscriptional regulation of NFATc1, which affects osteoclast differentiation through its functional methylation site (4249 A), is significantly influenced by RNA m6A methylation. After RANKL stimulation, low METTL14 expression lowers the NFATc1 m6A methylation level via the 4249 A site.

### 3.4 YTHDF2 negatively affects NFATc1's posttranscriptional regulation subsequent to METTL14's elevation of methylation levels in this gene

**FIGURE 4:**
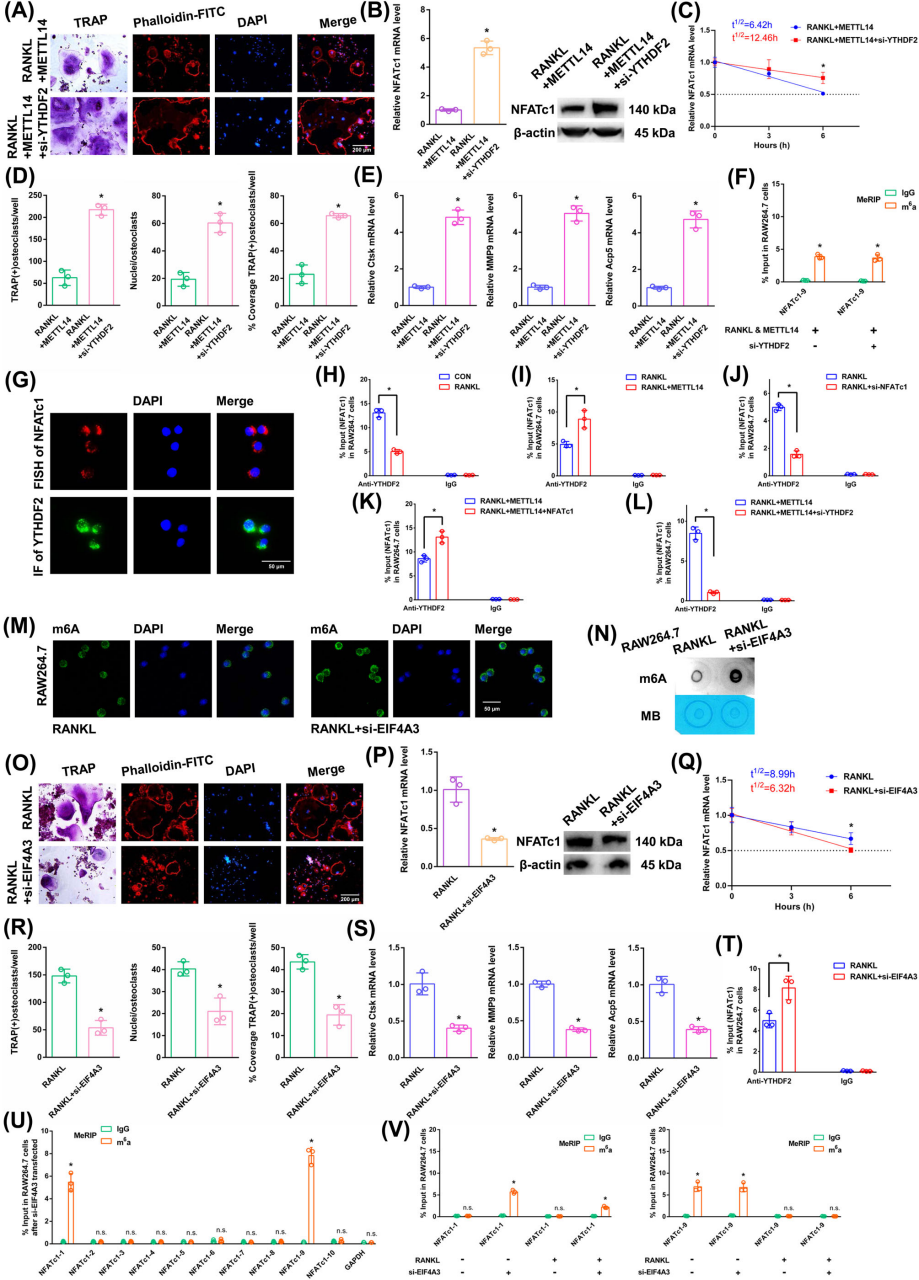
The effects of EIF4A3 on the posttranscriptional regulation of NFATc1 expression. (A) Osteoclast differentiation was assessed by TRAP staining and F‐actin band staining after stimulation with RANKL+METTL14+si‐YTHDF2. Scale bar: 200 µm. (B) The mRNA and protein expression levels of NFATc1 in RAW264.7 cells after stimulation with RANKL+METTL14+si‐YTHDF2. (C) The half‐life of NFATc1 mRNA was estimated by linear regression analysis after stimulation with RANKL+METTL14+si‐YTHDF2. (D) Histograms display the number, coverage rate and nuclei of TRAP‐positive osteoclasts after stimulation with RANKL+METTL14+si‐YTHDF2. (E) Relative expression levels of Ctsk, MMP9, and Acp5 in RAW264.7 cells after stimulation with RANKL+METTL14+si‐YTHDF2. (F) Enrichment of NFATc1‐9 segment after stimulation with RANKL+METTL14 or si‐YTHDF2 was detected by m6A‐RT‐qPCR. (G) Localization of NFATc1 and YTHDF2 were detected by FISH and immunofluorescence. (H–L) Enrichment rates of NFATc1 under various conditions were detected by RIP assay. (M, N) Immunofluorescence staining and m6A dot blot analysis show the overall m6A levels in RAW264.7 cells after stimulation with RANKL+si‐EIF4A3. Scale bar: 50 µm. (O) Osteoclast differentiation was assessed by TRAP staining and F‐actin band staining after stimulation with RANKL+si‐EIF4A3. Scale bar: 200 µm. (P) The mRNA and protein expression levels of NFATc1 in RAW264.7 cells after stimulation with RANKL+si‐EIF4A3. (Q) The half‐life of NFATc1 mRNA was estimated by linear regression analysis after stimulation with RANKL+si‐EIF4A3. (R) Histograms display the number, coverage rate and nuclei of TRAP‐positive osteoclasts after stimulation with RANKL+si‐EIF4A3. (S) Relative expression levels of Ctsk, MMP9, and Acp5 in RAW264.7 cells after stimulation with RANKL+si‐EIF4A3. (T) The enrichment rate of NFATc1 under RANKL+si‐EIF4A3 condition was detected by RIP assay. (U) Enrichment of ten NFATc1 segments between the anti‐m6A group and the anti‐IgG group after stimulation with si‐EIF4A3 was detected by m6A‐RT‐qPCR. (V) Enrichment of NFATc1‐1 and NFATc1‐9 segments after stimulation with RANKL or si‐EIF4A3 was detected by m6A‐RT‐qPCR. These data represent three independent experiments and are presented as the means ± SDs (*p < .05).

We next transfected si‐YTHDF2 to investigate the impact of “readers” on the expression of NFATc1 mRNA. The transgenic cell line was successfully constructed (Figure S1I). The YTHDF2 mRNA did not change in the alveolar bone of postmenopausal women with (P11–P20) or without osteoporosis (P1–P10) according to RT‐qPCR analysis (Figure S1J). Since other “readers” (YTHDF2 and IGF2BP2) were not involved in osteoclast differentiation, we set out to confirm the impact of YTHDF2 (Figure S3A–N). As demonstrated by the results of the aforementioned experiments, when we transfected si‐YTHDF2, the downregulation of YTHDF2 enhanced osteoclast differentiation after RANKL+METTL14 stimulation (Figure 4A,D,E). The NFATc1 expression was substantially increased after RANKL+METTL14+si‐YTHDF2 stimulation (Figure 4B). The half‐life of NFATc1 was extended after RANKL+METTL14+si‐YTHDF2 stimulation (Figure 4C). The NFATc1‐9 segment showed the same level of m6A methylation in both groups according to the m6A‐RT‐qPCR results (Figure 4F). Fluorescence in situ hybridization (FISH) and immunofluorescence staining exhibited that NFATc1 and YTHDF2 were both predominantly expressed in the cytoplasm (Figure 4G). RIP revealed that the YTHDF2 binding efficiency decreased after RANKL stimulation compared to that in the CON group. Moreover, the binding to the NFATc1 segment was greater in the RANKL+METTL14 group but lower in the RANKL+si‐NFATc1 group than in the RANKL group. In addition, the NFATc1 segment was elevated after NFATc1 overexpression compared to that in the RANKL+METTL14 group but was significantly decreased after si‐YTHDF2 stimulation (Figure 4H–L). These findings revealed that YTHDF2 and NFATc1 function by interacting. YTHDF2 contributed to the degradation of the NFATc1 mRNA after METTL14 increased its m6A methylation level.

### 3.5 The NFATc1 gene participates in the process by which osteoclast differentiation is inhibited by EIF4A3 knockdown

**FIGURE 5:**
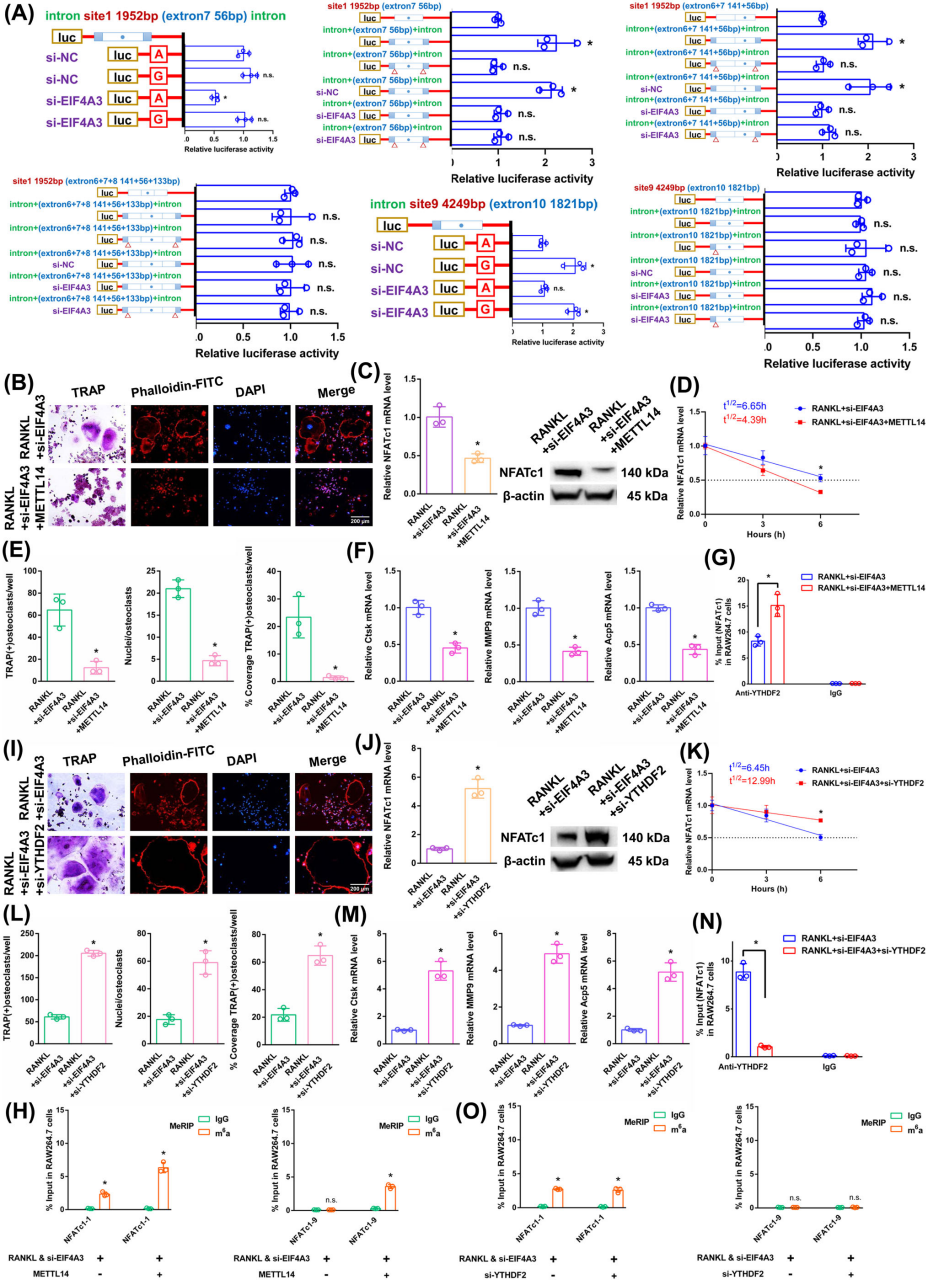
The effects of METTL14 and YTHDF2 on the posttranscriptional regulation of NFATc1 expression after RANKL+si‐EIF4A3 stimulation. (A) Validation of m6A modification sites through step‐by‐step mutation using luciferase reporters. First, we cloned the site at which NFATc1 expression was suppressed with its exon and 100 nt of flanking intron sequence into a luciferase reporter. Blue regions represent intron sequences derived from NFATc1. White areas indicate sequences from the exon sequences of NFATc1. “Δ” denotes a deletion of the specified introns (24 nt upstream of intron–exon junctions). (B) Osteoclast differentiation was assessed by TRAP staining and F‐actin band staining after stimulation with RANKL+si‐EIF4A3+METTL14. Scale bar: 200 µm. (C) The mRNA and protein expression levels of NFATc1 in RAW264.7 cells after stimulation with RANKL+si‐EIF4A3+METTL14. (D) The half‐life of NFATc1 mRNA was estimated by linear regression analysis after stimulation with RANKL+si‐EIF4A3+METTL14. (E) Histograms display the number, coverage rate and nuclei of TRAP‐positive osteoclasts after stimulation with RANKL+si‐EIF4A3+METTL14. (F) Relative expression levels of Ctsk, MMP9, and Acp5 in RAW264.7 cells after stimulation with RANKL+si‐EIF4A3+METTL14. (G) The enrichment rate of NFATc1 under RANKL+si‐EIF4A3+METTL14 condition was detected by RIP assay. (H) Enrichment of NFATc1‐1 and NFATc1‐9 segments after stimulation with RANKL+si‐EIF4A3 or METTL14 was detected by m6A‐RT‐qPCR. (I) Osteoclast differentiation was assessed by TRAP staining and F‐actin band staining after stimulation with RANKL+si‐EIF4A3+si‐YTHDF2. Scale bar: 200 µm. (J) The mRNA and protein expression levels of NFATc1 in RAW264.7 cells after stimulation with RANKL+si‐EIF4A3+si‐YTHDF2. (K) The half‐life of NFATc1 mRNA was estimated by linear regression analysis after stimulation with RANKL+si‐EIF4A3+si‐YTHDF2. (L) Histograms display the number, coverage rate and nuclei of TRAP‐positive osteoclasts after stimulation with RANKL+si‐EIF4A3+si‐YTHDF2. (M) Relative expression levels of Ctsk, MMP9, and Acp5 in RAW264.7 cells after stimulation with RANKL+si‐EIF4A3+si‐YTHDF2. (N) The enrichment rate of NFATc1 in the RANKL+si‐EIF4A3+si‐YTHDF2 group was detected by RIP assay. (O) Enrichment of NFATc1‐1 and NFATc1‐9 segments after stimulation with RANKL+si‐EIF4A3 or si‐YTHDF2 was detected by m6A‐RT‐qPCR. These data represent three independent experiments and are presented as the means ± SDs (*p < .05).

In RAW264.7 cells, we knocked down the core EJC factor EIF4A3. The transgenic cell line was successfully constructed (Figure S1K). The EIF4A3 mRNA did not change in the alveolar bone of postmenopausal women with (P11–P20) or without osteoporosis (P1–P10) according to RT‐qPCR analysis (Figure S1L). After RANKL+siEIF4A3 stimulation, the overall m6A methylation level was strongly increased, as shown by dot blot and immunofluorescence assays (Figure 4 M,N). As demonstrated by the results of the aforementioned experiments, when we transfected si‐EIF4A3, the downregulation of EIF4A3 decreased osteoclast differentiation to a certain extent after RANKL stimulation (Figure 4O,R,S). The NFATc1 expression was substantially decreased after RANKL+si‐EIF4A3 stimulation (Figure 4P). The half‐life of NFATc1 was shortened to a certain extent after RANKL+si‐EIF4A3 stimulation (Figure 4Q). RIP assays revealed that the YTHDF2 binding efficiency increased after RANKL+si‐EIF4A3 stimulation compared to that in the RANKL group (Figure 4T). After si‐EIF4A3 stimulation, the m6A‐RT‐qPCR results showed that the previously high level of the m6A methylated NFATc1‐9 segment was maintained. However, after EIF4A3 knockdown, the previously low level of the m6A methylated NFATc1‐1 segment was significantly increased (Figure 4U). After RANKL stimulation, the levels of both m6A methylated segments (NFATc1‐1 and NFATc1‐9) were significantly decreased (Figure 4V). Next, m6A modification sites 1 and 9 (in exons 7 and 10) were confirmed via step‐by‐step mutation of reporter plasmids. In particular, different mutant NFATc1 fragments were inserted into the luciferase reporters individually (site 1: 1952A‐G; site 9: 4249A‐G). These findings revealed that site 1 was an effective m6A methylation site after EIF4A3 knockdown. Site 9 was not affected by EIF4A3 (Figure 5A). We next designed another luciferase reporter assay to prove the protective function of EIF4A3, which prevents m6A deposition in average length internal exons but not in terminal or long internal exons. The results showed that EIF4A3 protected the methylation site from hypermethylation through the intron‐exon binding region when the length of the exon fragment at the methylation site was 50 nt (exon 7, 56 nt) to 200 nt (exons 6+7, 197 nt), and the protective effect disappeared when the length of the exon fragment at the methylation site reached 300 nt (exons 6+7+8, 330 nt) (Figure 5A).

### 3.6 The NFATc1 gene participates in the process by which osteoclast differentiation is strongly inhibited by si‐EIF4A3+METTL14

The above results proved that si‐EIF4A3 or METTL14 inhibited osteoclast differentiation to a certain extent. In this study, we both knocked down EIF4A3 and overexpressed METTL14 in RAW264.7 cells. As demonstrated by the results of the aforementioned experiments, when we transfected si‐EIF4A3+METTL14, osteoclast differentiation was strongly decreased after RANKL stimulation (Figure 5B,E,F). The NFATc1 expression was substantially decreased after RANKL+si‐EIF4A3+METTL14 stimulation (Figure 5C). The half‐life of NFATc1 was markedly shortened after RANKL+si‐EIF4A3+METTL14 stimulation (Figure 5D). RIP assays revealed that the YTHDF2 binding efficiency increased after RANKL+si‐EIF4A3+METTL14 stimulation compared to that in the RANKL+si‐EIF4A3 group (Figure 5G). According to the m6A‐RT‐qPCR results, METTL14 increased the levels of the NFATc1‐9 segment and the other segment (NFATc1‐1) after RANKL+si‐EIF4A3 stimulation (Figure 5H). Taken together, these findings indicate that METTL14 regulates m6A methylation at all sites. EIF4A3 protection is adequate to prevent m6A deposition in average‐length internal exons but not in terminal or long internal exons.

### 3.7 YTHDF2 negatively affects the posttranscriptional regulation of NFATc1 expression following the elevation of its methylation levels by si‐EIF4A3

The aforementioned results proved that YTHDF2 negatively affects the posttranscriptional regulation of NFATc1 expression following the elevation of its methylation levels by METTL14. In this study, we knocked down both EIF4A3 and YTHDF2 in RAW264.7 cells. As demonstrated by the results of the aforementioned experiments, when we transfected si‐EIF4A3+si‐YTHDF2, osteoclast differentiation was strongly increased after RANKL stimulation (Figure 5I,L,M). The NFATc1 expression was markedly increased after RANKL+si‐EIF4A3+si‐YTHDF2 stimulation (Figure 5J). The half‐life of NFATc1 was substantially prolonged after RANKL+si‐EIF4A3+si‐YTHDF2 stimulation (Figure 5K). RIP assays revealed that the YTHDF2 binding efficiency decreased after RANKL+si‐EIF4A3+si‐YTHDF2 stimulation compared with that in the RANKL+si‐EIF4A3 group (Figure 5N). The NFATc1‐9 segment and the other segment (NFATc1‐1) showed the same level of m6A methylation in both groups according to the m6A‐RT‐qPCR results (Figure 5O). Taken together, these findings led to the conclusion that YTHDF2 promoted the degradation of NFATc1 transcripts without a site restriction.

### 3.8 The NFATc1 gene participates in the process by which osteoclast differentiation is inhibited by RBM8A knockdown

**FIGURE 6:**
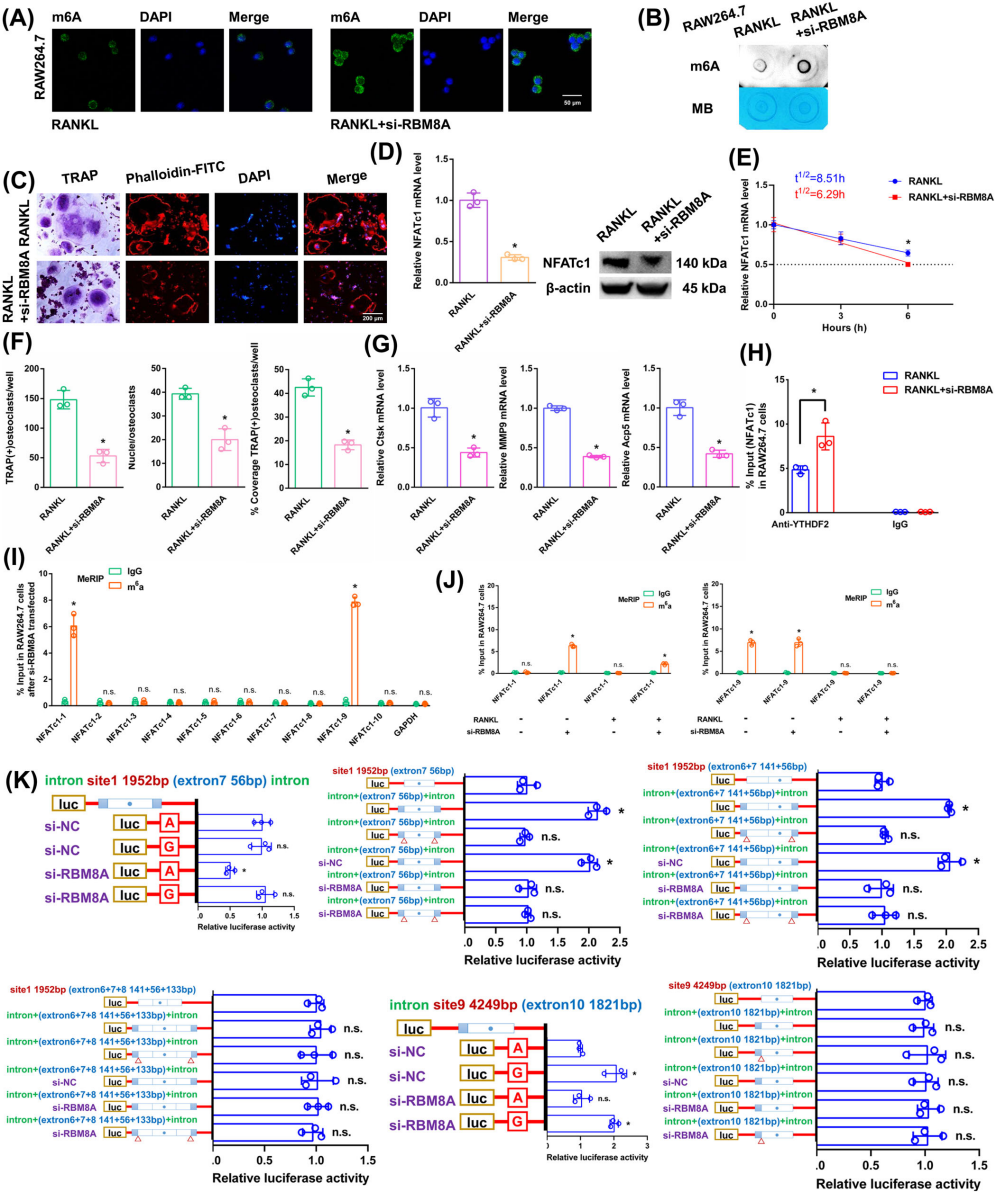
The effects of RBM8A on the posttranscriptional regulation of NFATc1 expression. (A, B) Immunofluorescence staining and m6A dot blot analysis show the overall m6A levels in RAW264.7 cells after stimulation with RANKL+si‐RBM8A. Scale bar: 50 µm. (C) Osteoclast differentiation was assessed by TRAP staining and F‐actin band staining after stimulation with RANKL+si‐RBM8A. Scale bar: 200 µm. (D) The mRNA and protein expression levels of NFATc1 in RAW264.7 cells after stimulation with RANKL+si‐RBM8A. (E) The half‐life of NFATc1 mRNA was estimated by linear regression analysis after stimulation with RANKL+si‐RBM8A. (F) Histograms display the number, coverage rate and nuclei of TRAP‐positive osteoclasts after stimulation with RANKL+si‐RBM8A. (G) Relative expression levels of Ctsk, MMP9, and Acp5 in RAW264.7 cells after stimulation with RANKL+si‐RBM8A. (H) The enrichment rate of NFATc1 under RANKL+si‐RBM8A condition was detected by RIP assay. (I) Enrichment of ten NFATc1 segments between the anti‐m6A group and the anti‐IgG group after stimulation with si‐RBM8A was detected by m6A‐RT‐qPCR. (J) Enrichment of NFATc1‐1 and NFATc1‐9 segments after stimulation with RANKL or si‐RBM8A was detected by m6A‐RT‐qPCR. (K) Validation of m6A modification sites through step‐by‐step mutation using luciferase reporters. Initially, we cloned the sites of the exon and flanking intron 100 nt sequences from NFATc1 where expression was suppressed into a luciferase reporter. Blue regions represent intron sequences derived from NFATc1. White regions indicate sequences from the exon region of NFATc1. “Δ” denotes deletion of the specified introns (24 nt upstream of intron‐exon junctions). These data represent three independent experiments and are presented as the means ± SDs (*p < .05).

In RAW264.7 cells, we knocked down the core EJC factor RBM8A. The transgenic cell line was successfully constructed (Figure S1M). The RBM8A mRNA did not change in the alveolar bone of postmenopausal women with (P11‐P20) or without osteoporosis (P1–P10) according to RT‐qPCR analysis (Figure S1N). After RANKL+si‐RBM8A stimulation, the overall m6A methylation level was remarkably increased, as shown by dot blot and immunofluorescence assays (Figure 6A,B). As demonstrated by the results of the aforementioned experiments, when we transfected si‐RBM8A, the downregulation of RBM8A decreased osteoclast differentiation to a certain extent after RANKL stimulation (Figure 6C,F,G). The NFATc1 expression was substantially decreased after RANKL+si‐RBM8A stimulation (Figure 6D). The half‐life of NFATc1 was shortened to a certain extent after RANKL+si‐RBM8A stimulation (Figure 6E). RIP assays revealed that the YTHDF2 binding efficiency increased after RANKL+si‐RBM8A stimulation compared to that in the RANKL group (Figure 6H). After si‐RBM8A stimulation, the m6A‐RT‐qPCR results showed that the previously high level of the m6A methylated NFATc1‐9 segment was maintained. However, after RBM8A knockdown, the previously low level of the m6A methylated NFATc1‐1 segment was significantly increased (Figure 6I). After RANKL stimulation, the levels of both m6A methylated segments (NFATc1‐1 and NFATc1‐9) were significantly decreased (Figure 6J). Next, m6A modification sites 1 and 9 (in exons 7 and 10) were confirmed via step‐by‐step mutation of the reporter plasmids. In particular, different mutant NFATc1 fragments were inserted into the luciferase reporters individually (site 1: 1952A‐G; site 9: 4249A‐G). These findings indicated that site 1 was an effective m6A methylation site after RBM8A knockdown. Site 9 was not affected by RBM8A (Figure 6K). We next designed another luciferase reporter assay to prove the protective function of RBM8A, which prevents m6A deposition in average length internal exons but not in terminal or long internal exons. The results showed that RBM8A protected the methylation site from hypermethylation through the intron‐exon binding region when the length of the exon fragment at the methylation site was 50 nt (exon 7, 56 nt) to 200 nt (exons 6+7, 197 nt), and the protective effect disappeared when the length of the exon fragment at the methylation site reached 300 nt (exons 6+7+8, 330 nt) (Figure 6K).

### 3.9 The NFATc1 gene participates in the process by which osteoclast differentiation is strongly inhibited by si‐RBM8A+METTL14

**FIGURE 7:**
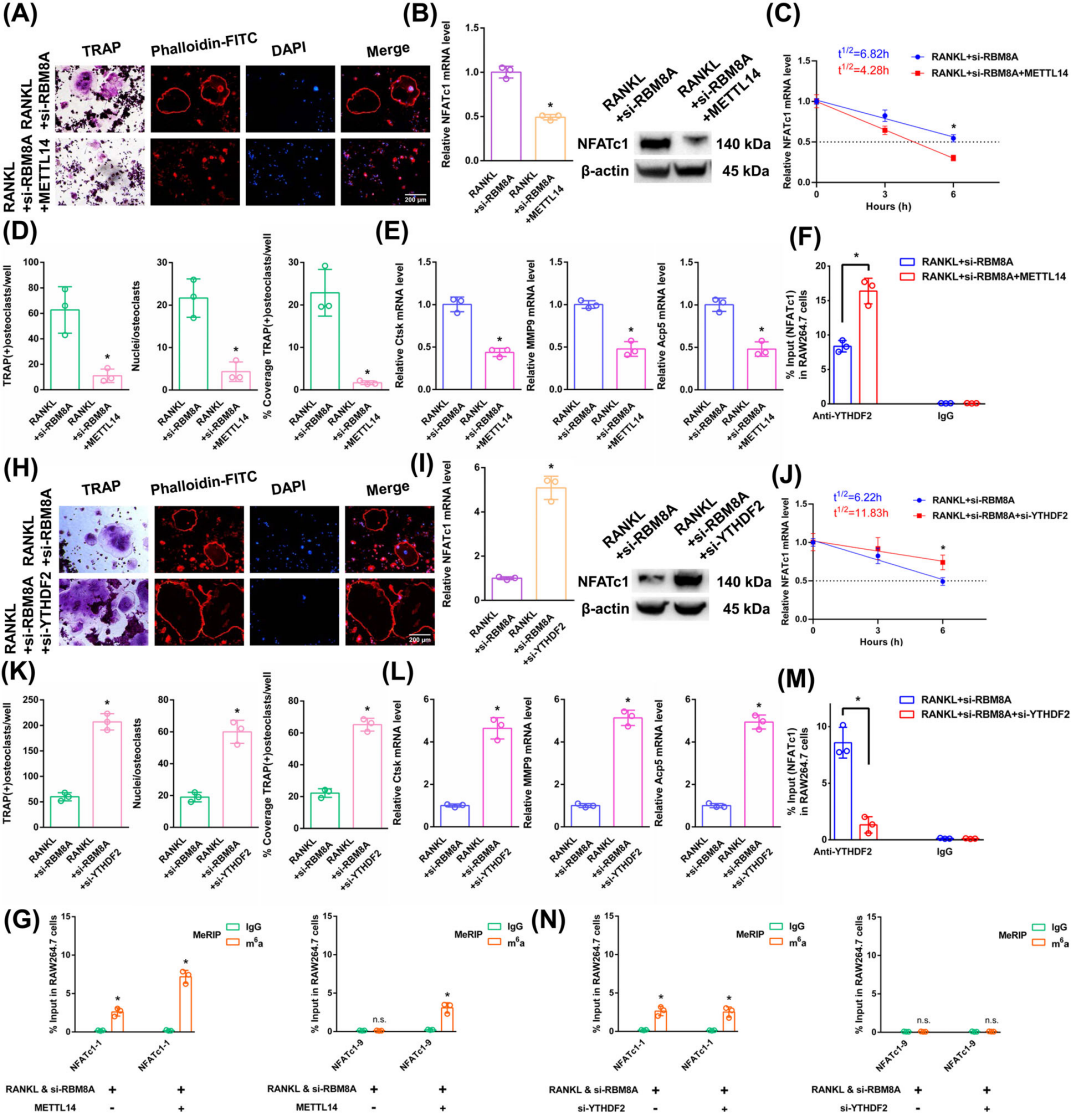
The effects of METTL14 and YTHDF2 on the posttranscriptional regulation of NFATc1 after RANKL+si‐RBM8A stimulation. (A) Osteoclast differentiation was detected by TRAP staining and F‐actin band staining after stimulation with RANKL+si‐RBM8A+METTL14. Scale bar: 200 µm. (B) The mRNA and protein expression levels of NFATc1 in RAW264.7 cells after stimulation with RANKL+si‐RBM8A+METTL14. (C) The half‐life of NFATc1 mRNA was estimated by linear regression analysis after stimulation with RANKL+si‐RBM8A+METTL14. (D) Histograms display the number, coverage rate and nuclei of TRAP‐positive osteoclasts after stimulation with RANKL+si‐RBM8A+METTL14. (E) The relative expression levels of Ctsk, MMP9, and Acp5 in RAW264.7 cells after stimulation with RANKL+si‐RBM8A+METTL14. (F) The enrichment rate of NFATc1 in the RANKL+si‐RBM8A+METTL14 group was detected by RIP assay. (G) The enrichment of NFATc1‐1 and NFATc1‐9 segments after stimulation with RANKL+si‐RBM8A or METTL14 was detected by m6A‐RT‐qPCR. (H) Osteoclast differentiation was detected by TRAP staining and F‐actin band staining after stimulation with RANKL+si‐RBM8A+si‐YTHDF2. Scale bar: 200 µm. (I) The mRNA and protein expression levels of NFATc1 in RAW264.7 cells after stimulation with RANKL+si‐RBM8A+si‐YTHDF2. (J) The half‐life of NFATc1 mRNA was estimated by linear regression analysis after stimulation with RANKL+si‐RBM8A+si‐YTHDF2. (K) Histograms display the number, coverage rate and nuclei of TRAP‐positive osteoclasts after stimulation with RANKL+si‐RBM8A+si‐YTHDF2. (L) The relative expression levels of Ctsk, MMP9, and Acp5 in RAW264.7 cells after stimulation with RANKL+si‐RBM8A+si‐YTHDF2. (M) The enrichment rate of NFATc1 in the RANKL+si‐RBM8A+si‐YTHDF2 group was detected by RIP assay. (N) The enrichment of NFATc1‐1 and NFATc1‐9 segments after stimulation with RANKL+si‐RBM8A or si‐YTHDF2 was detected by m6A‐RT‐qPCR. These data represent three independent experiments and are presented as the means ± SDs (*p < .05).

The above results proved that si‐RBM8A or METTL14 inhibited osteoclast differentiation to a certain extent. In this study, we both knocked down RBM8A and overexpressed METTL14 in RAW264.7 cells. As demonstrated by the results of the aforementioned experiments, when we transfected si‐RBM8A+METTL14, osteoclast differentiation was strongly decreased after RANKL stimulation (Figure 7A,D,E). The NFATc1 expression was substantially decreased after RANKL+si‐RBM8A+METTL14 stimulation (Figure 7B). The half‐life of NFATc1 was markedly shortened after RANKL+si‐RBM8A+METTL14 stimulation (Figure 7C). RIP assays revealed that the YTHDF2 binding efficiency increased after RANKL+si‐RBM8A+METTL14 stimulation compared to that in the RANKL+si‐RBM8A group (Figure 7F). According to the m6A‐RT‐qPCR results, METTL14 increased the levels of the NFATc1‐9 segment and the other segment (NFATc1‐1) after RANKL+si‐RBM8A stimulation (Figure 7G). Taken together, these findings indicate that METTL14 regulates m6A methylation at all sites. RBM8A protection is adequate to prevent m6A deposition in average‐length internal exons but not in terminal or long internal exons.

### 3.10 YTHDF2 negatively affects the posttranscriptional regulation of NFATc1 expression following the elevation of its methylation levels by si‐RBM8A

The aforementioned results proved that YTHDF2 negatively affects the posttranscriptional regulation of NFATc1 expression following the elevation of its methylation levels by METTL14. In this study, we knocked down both RBM8A and YTHDF2 in RAW264.7 cells. As demonstrated by the results of the aforementioned experiments, when we transfected si‐RBM8A+si‐YTHDF2, osteoclast differentiation was strongly increased after RANKL stimulation (Figure 7H,K,L). The NFATc1 expression was markedly increased after RANKL+si‐RBM8A+si‐YTHDF2 stimulation (Figure 7I). The half‐life of NFATc1 was substantially prolonged after RANKL+si‐RBM8A+si‐YTHDF2 stimulation (Figure 7J). RIP assays revealed that the YTHDF2 binding efficiency decreased after RANKL+si‐RBM8A+si‐YTHDF2 stimulation compared to that in the RANKL+si‐RBM8A group (Figure 7M). The NFATc1‐9 segment and the other segment (NFATc1‐1) showed the same level of m6A methylation in both groups according to the m6A‐RT‐qPCR results (Figure 7N). Taken together, these findings led to the conclusion that YTHDF2 promoted the degradation of NFATc1 transcripts without a site restriction. Consistently, si‐YTHDF2 rescued the accelerated degradation of YTHDF2 target transcripts upon the transfection of si‐EIF4A3 or si‐RBM8A, which are osteoclast differentiation‐related genes (Figures S4 and S5). Since we could not find any connections between the EJCs and the methyltransferase complex, steric hindrance from the EJCs may account for the suppression phenomenon of m6A methylation (Figure S6). Moreover, the function of another EJC member (MAGOH) was confirmed. The same trend was observed after si‐MAGOH stimulation as for EIF4A3 and RBM8A (Figure S7).

### 3.11 Loss of EIF4A3/RBM8A clearly rescues bone loss to a certain extent

**FIGURE 8:**
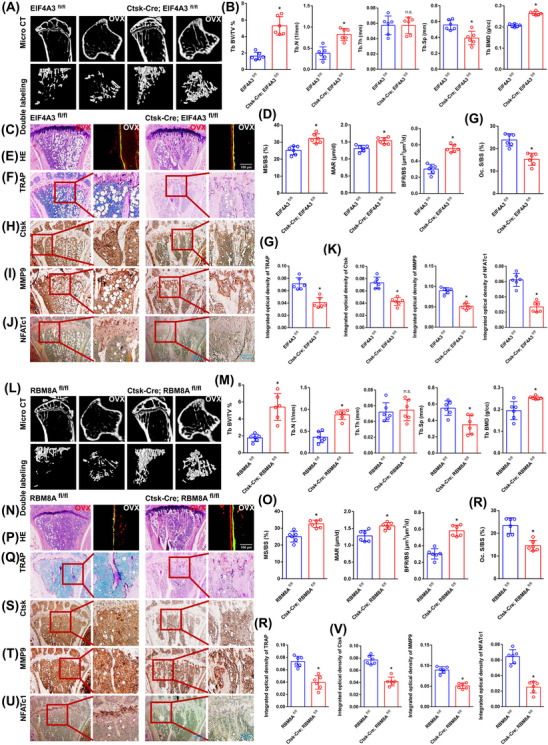
The effects of EIF4A3/RBM8A in vivo. In vivo study was conducted using conditional EIF4A3 or RBM8A knockout mice. (A) Eight weeks after OVX, micro‐CT was used to compare bone histological parameters between the EIF4A3^fl/fl^ group and the Ctsk‐Cre; EIF4A3^fl/fl^ group. (B) Histograms comparing Tb.N, Tb BV/TV, Tb.Sp, Tb.Th, and Tb.BMD between the two groups. (C, D) Representative images of dynamic cortical bone histomorphometry quantified by MS/BS, MAR, and BFR/BS. Scale bar: 100 µm. (E) Comparison of bone histological parameters between the two groups using H&E staining. Scale bars: 200 µm. (F) Evaluation of osteoclast differentiation and bone resorption using TRAP staining. Scale bars: 200 and 50 µm. (G) Histograms comparing Oc.S/BS and the integrated optical density of TRAP staining between the two groups. (H–J) Immunohistochemical staining for Ctsk, MMP9 and NFATc1 to evaluate osteoclast differentiation and bone resorption. Scale bars: 200 and 50 µm. (K) Histograms comparing the integrated optical densities of Ctsk, MMP9, and NFATc1 between the two groups. (L) Eight weeks after OVX, micro‐CT was used to compare bone histological parameters between the RBM8A^fl/fl^ group and the Ctsk‐Cre; RBM8A^fl/fl^ group. (M) Histograms comparing Tb.N, Tb BV/TV, Tb.Sp, Tb.Th, and Tb.BMD between the two groups. (N, O) Representative images of dynamic cortical bone histomorphometry quantified by MS/BS, MAR, and BFR/BS. Scale bar: 100 µm. (P) Comparison of bone histological parameters between the two groups using H&E staining. Scale bars: 200 µm. (Q) Evaluation of osteoclast differentiation and bone resorption using TRAP staining. Scale bars: 200 and 50 µm. (R) Histograms comparing Oc.S/BS and the integrated optical density of TRAP staining between the two groups. (S–U) Immunohistochemical staining for Ctsk, MMP9, and NFATc1 to evaluate osteoclast differentiation and bone resorption. Scale bars: 200 and 50 µm. (V) Histograms comparing the integrated optical densities of Ctsk, MMP9, and NFATc1 between the two groups. These data represent six independent experiments and are presented as the means ± SDs (*p < .05).

Bone histological parameters were measured using micro‐CT. After 8 weeks, the OVX mice from the EIF4A3^fl/fl^ group showed distinct bone loss compared with those in the Ctsk‐Cre; EIF4A3^fl/fl^ group. Bone loss was rescued in the Ctsk‐Cre; EIF4A3^fl/fl^ group to a certain extent (Figure 8A). Compared with the EIF4A3^fl/fl^ group, the Ctsk‐Cre; EIF4A3^fl/fl^ group exhibited observably increased trabecular number (Tb.N), trabecular BMD and trabecular bone volume/total volume (BV/TV) but significantly decreased trabecular separation (Tb.Sp) (Figure 8B). Double fluorescence‐labeled sections exhibited that the EIF4A3^fl/fl^ group had remarkably less cortical bone. Compared to the EIF4A3^fl/fl^ group, the Ctsk‐Cre; EIF4A3^fl/fl^ group displayed a remarkable rescue phenomenon to a certain extent (Figure 8C). The MAR, bone formation rate/bone surface (BFR/BS) and mineralizing surface/bone surface (MS/BS) exhibited the same trend between the two groups (Figure 8D). The same trend was confirmed by H&E staining (Figure 8E). As expected from TRAP staining, the EIF4A3^fl/fl^ group revealed a significantly increased TRAP‐positive osteoclast number. A clear inhibitory effect on osteoclast differentiation was observed in the Ctsk‐Cre; EIF4A3^fl/fl^ group (Figure 8F). Several significant differences in the integrated optical density of TRAP staining and the osteoclast surface/bone surface (Oc. S/BS) between the two groups are presented in the histograms (Figure 8G). Immunohistochemical staining for Ctsk, MMP9 and NFATc1 exhibited the same trend between the two groups (Figure 8H–J). Several significant differences in the integrated optical densities of MMP9, Ctsk and NFATc1 between the two groups are presented in the histograms (Figure 8K). As demonstrated by the results of the aforementioned experiments (micro‐CT, dynamic histomorphometry of double fluorescence labeling, H&E staining, TRAP staining and immunohistochemistry assays), the same trend was observed between the RBM8A^fl/fl^ group and the Ctsk‐Cre; RBM8A^fl/fl^ group (Figure 8L–V). EJCs can raise the level of m6A methylation of NFATc1 and degrade its mRNA, thereby inhibiting osteoclast differentiation and preserving bone mass. These results will be helpful for identifying potential molecular targets for the therapy of osteoporosis. In particular, it will be useful to inhibit abnormal osteoclast differentiation in postmenopausal osteoporosis.

## 4 DISCUSSION

Recent studies showed the molecular mechanisms between m6A modification and osteoclast differentiation. Deng found that the expression of METTL14 was decreased in postmenopausal patients with osteoporosis, and overexpression of METTL14 could stabilize GPX4 mRNA and inhibit osteoclast differentiation and bone absorption.^26^ In addition, Wang found that METTL14 can correct osteoporosis by increasing the m6A methylation level of SIRT1.^27^ FTO and WTAP correct osteoporosis by inhibiting osteoclast differentiation.^28^, ^29^ Li found that METTL3‐YTHDF2 affected osteoclast differentiation and bone resorptive function by regulating ATP6V0D2 mRNA degradation and TRAF6 mRNA nuclear export.^30^ EGR1 causes osteoclast differentiation in osteoporosis by upregulating CHI3L1 levels through METTL3.^31^ In addition, YTHDF1 promotes osteoclast differentiation by regulating TNFRSF11A mRNA stabilization and endoplasmic reticulum stress.^32^

This work clarifies the process of osteoclast differentiation from an RNA methylation standpoint. In particular, RNA m6A methylation plays a crucial part in the posttranscriptional regulation of NFATc1, which influences osteoclast differentiation via its functional methylation site (4249 A). Low METTL14 expression reduces the NFATc1 m6A methylation level via the 4249 A site after RANKL stimulation. Downstream, YTHDF2 reduced the preservation of the NFATc1 mRNA by binding to m6A modification sites. This finding suggested that NFATc1 expression increased after RANKL stimulation. In this study, the NFATc1 m6A methylation level was strongly correlated with osteoclast‐induced bone resorption. The NFATc1 gene's functional m6A methylation site is 4249 A. In previous research, m6A modifies the local structure of mRNAs and lncRNAs to promote the binding of heterogeneous nuclear ribonucleoprotein C. The name “m6A switch” refers to the mechanism that controls RNA protein interactions via m6A‐dependent RNA structural remodeling.^33^ Additionally, our previous study revealed a similar phenomenon. By acting on the m6A functional site in circ_0008542, METTL3 causes the target gene RANK to be highly expressed and initiates osteoclast‐mediated bone absorption.^12^ Furthermore, our team's previous study also revealed a clear correlation between reduced osteoclast bone resorption and the elevated NFATc1 m6A methylation caused by zoledronic acid.^13^ In this study, we defined 4249 A as the “m6A switch” based on the previous conclusions. There is a close relationship between osteoclast differentiation and bone resorption, and this “m6A switch” at NFATc1 4249 A. It appears that METTL14 and YTHDF2 are nodes that regulate the “m6A switch” in varying degrees. Our study indicates that different key molecules within the same signaling pathway collectively regulate osteoclasts’ m6A methylation‐related bone resorption. This study provides an innovative theory for the precise regulation of osteoclast differentiation through functional m6A methylation sites in osteoporosis treatment.

The previously discovered m6A effector proteins can be roughly classified into three groups: “writers”, which catalyze m6A methylation; “readers”, which selectively bind m6A; and “erasers”, which reverse m6A methylation.^34^, ^35^, ^36^, ^37^, ^38^, ^39^, ^40^, ^41^, ^42^, ^43^ Here, we showed that during osteoclast differentiation, EJCs belong to a novel class of m6A regulators called “shields”, which generally suppress m6A sites in genes related to osteoclast differentiation, particularly the NFATc1 gene. EJCs seem to be major regulators of m6A deposition in the NFATc1 gene, mediating several important aspects of global m6A epitranscriptome specificity, such as methylation selectivity for transcripts with long internal exons and m6A enrichment in the last exons near stop codons. In this study, we detected the function of two core EJC factors, EIF4A3 and RBM8A, during osteoclast differentiation. Our results proved that m6A deposition in internal exons can be prevented by EIF4A3‐ or RBM8A‐mediated, but not in terminal exons. The length of exons in transcripts is a functionally significant component of the posttranscriptional regulation of gene expression. The suppressed m6A sites in the internal exons are located in much shorter exons (50–200 nt). However, the inhibitory effect disappeared when the length of the exon fragment at the methylation site reached 300 nt. Specifically, ten potential m6A methylation sites are present in the NFATc1 gene, and NFATC1‐9 4249 A is a functional methylation site residing within the 3′ UTR of the gene, according to m6A‐RT‐qPCR and luciferase reporter assays. Three methylation sites (1952 A, 2164 A, and 2386 A) were identified in the CDS region of the gene, and m6A‐RT‐qPCR and luciferase reporter assays indicated that only the methylation level of 1952A increased after EIF4A3/RBM8A knockdown. In addition, 1952 A is located in exon‐7 of the NFATc1 gene, with an exon‐7 fragment length of 56 nt. We then found that EIF4A3/RBM8A protects this site from hypermethylation when the exon length at both ends of 1952 A is within 50–200 nt (exon 7, 56 nt or exon 6+7, 141+56 nt), but the “shield effect” of EIF4A3/RBM8A disappears when the exon length at both ends is extended to 300 nt (exon 6+7+8, 141+56+133 nt). Moreover, the functional methylation site 4249 A in NFATc1‐9 was not regulated by EIF4A3/RBM8A. These findings prove that EJCs can protect the m6A methylation site in the CDS region from hypermethylation and degradation, but the range of sequences protected by EJCs is less than 200 nt, and the methylation site in the 3′ UTR is not protected by EJCs. These findings suggested that these pretranslational osteoclast differentiation‐related mRNAs are stably bound by EJCs at precisely spaced intervals. Downstream, METTL14 regulates NFATc1 m6A methylation at all sites, both at internal exons and terminal exons. YTHDF2 induced the degradation of relevant transcripts. Similarly, other genes involved in osteoclast differentiation are also regulated by EIF4A3 or RBM8A. These target transcripts are subsequently sequentially disposed of by METTL14 and YTHDF2.

By raising the NFATc1 gene's m6A methylation level to inhibit osteoclasts 8 weeks after OVX, conditional knockout of EIF4A3 or RBM8A rescued bone loss to a certain extent. This finding is in line with the in vitro experiments. It is a new molecular mechanism during osteoclast differentiation. Osteoclast differentiation is inhibited by altering the NFATc1 gene's m6A methylation level, thereby preserving bone mass and restoring a normal bone remodeling status. In addition, both METTL14 and EJCs are involved in RANKL‐induced osteoclast differentiation to a certain extent. The results of this study will be helpful for identifying potential molecular targets for the treatment of osteoporosis.

EJCs are composed of two core proteins, EIF4A3 and RBM8A, as well as other auxiliary proteins. We have only verified their role in osteoclast differentiation. The role of other molecules has not been investigated. In addition, this study verified the regulatory effect of EJCs on the m6A methylation level of NFATc1's exon fragments. For other genes that affect osteoclast differentiation, we only found that their half‐lives were regulated by EJCs; whether the “shield effect” of EJCs in other genes, cells and diseases has not been proven. Our team has been engaged in the role of m6A methylation modification in osteoclast differentiation for a long time. In the future, we will further study the mechanism of the specific distribution of m6A methylation modification.

## 5 CONCLUSION

EJCs selectively protect the m6A methylation sites of the NFATc1 gene. When the methylation sites are located within short exon fragments (50–200 nt), EJCs prevent their hypermethylation and degradation through the “shield effect”; when the methylation sites are located in the 3′ UTR region or long exon fragments (greater than 300 nt), the “shield effect” disappears. Downstream, YTHDF2 induced the degradation of hypermethylation NFATc1 transcripts without site restriction. EJCs act as “shields” to regulate the m6A region selectivity of the NFATc1 gene, thereby determining the characteristics of m6A distribution in the gene. Importantly, EJCs can raise the level of m6A methylation of NFATc1 and degrade its mRNA, thereby inhibiting osteoclast differentiation and preserving bone mass. These results will be helpful for identifying potential molecular targets for osteoporosis treatment.

## AUTHOR CONTRIBUTIONS

Bao Sun, Jin‐Gang Yang, and Zhe Wang contributed equally to this work as co‐first authors. Bao Sun, Jin‐Gang Yang, Zhe Wang, and Wei Wang performed most of the experiments. Wei Wang, Ping Zhang, Ya‐qin Zhu, and Jiang Li designed the experiments. Wei Wang wrote the manuscript. Xing Li and Sheng‐nan Liu assisted with the construction of the animal model and the in vitro experiments. Zheng Wang and Wei Feng assisted with the data analysis.

## CONFLICT OF INTEREST STATEMENT

The authors declare no conflict of interest.

## ETHICS STATEMENT

Ethical approval for the use of all clinical samples was granted by the Ethics Committee of the School of Stomatology, Nanjing Medical University, with written informed consent obtained from every participant. Animal experimentation conducted within this study adhered to the “Guidelines for Animal Experimentation” established by the School of Stomatology, Nanjing Medical University. All experimental protocols involving animals were reviewed and approved by the Laboratory Animal Care and Use Committees of the hospital.

## Supporting information



Supporting Information

Supporting Information

Supporting Information

## Data Availability

The data supporting the findings of this study are available from the corresponding author upon reasonable request.
