# Supplementary material for: Exon junction complexes regulate osteoclast‐induced bone resorption by influencing the NFATc1 m6A distribution through the “shield effect”
Source: Clin Transl Med. 2025 Mar 6;15(3):e70266. doi: 10.1002/ctm2.70266 (PMC11885169; doi:10.1002/ctm2.70266)
Supplement: Supplementary file 1 — Supporting Information [file CTM2-15-e70266-s003.docx]

**Supplementary figures and tables**

**
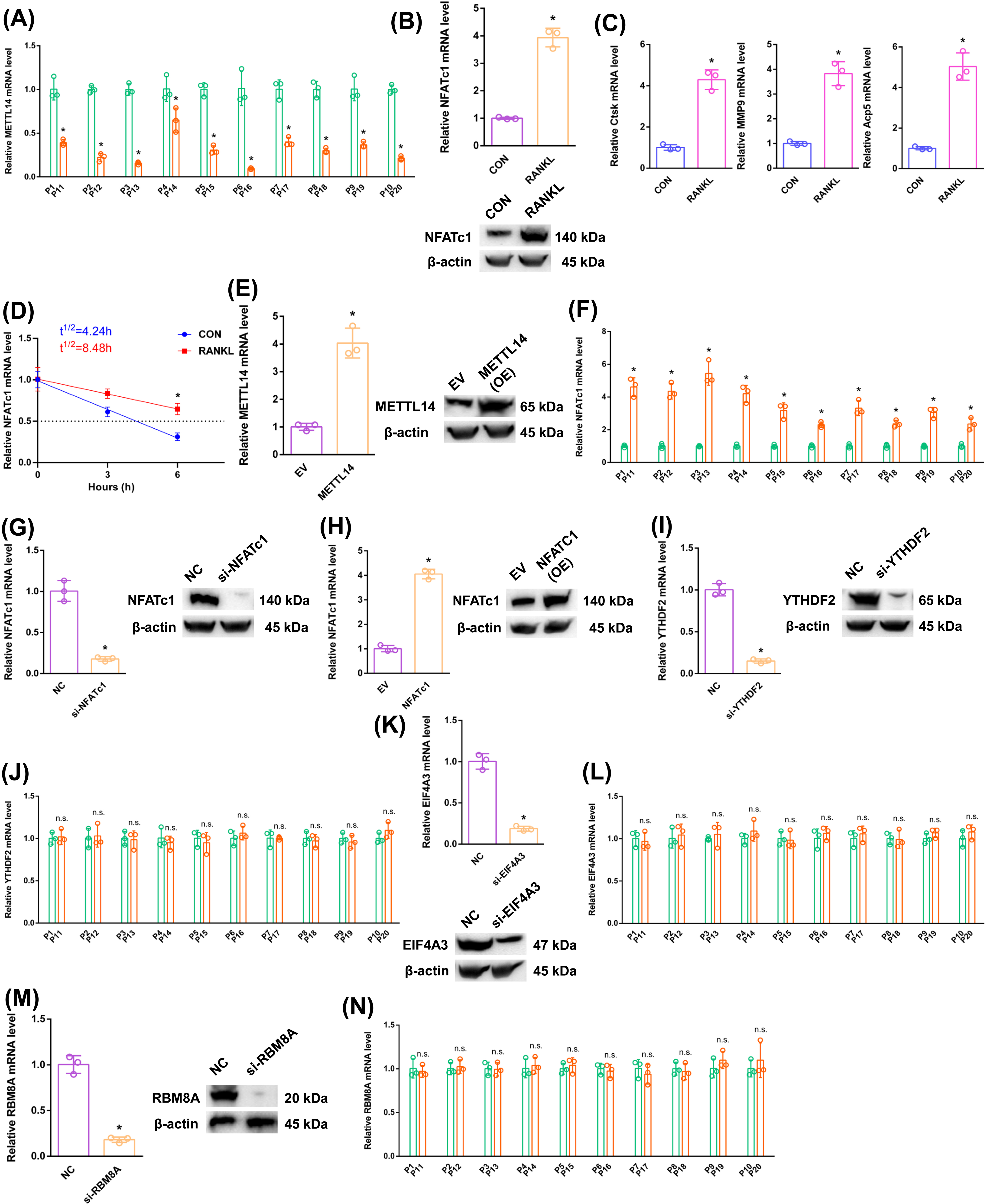
**

**Fig. S1. (A)** METTL14 mRNA expression levels in human bone samples from postmenopausal women with or without osteoporosis were detected using RT-qPCR. **(B)** After RANKL stimulation, the NFATc1 mRNA and protein expression levels in RAW264.7 cells were detected using RT-qPCR and western blotting, respectively. **(C)** Relative expression levels of Ctsk, MMP9 and Acp5 in RAW264.7 cells after RANKL stimulation. **(D)** After RANKL stimulation, the NFATc1 mRNA half-life was estimated via linear regression analysis. **(E)** After METTL14 was transfected into RAW264.7 cells, the METTL14 mRNA and protein expression levels were detected using RT-qPCR and western blotting, respectively. **(F)** The NFATc1 mRNA expression levels in human bone samples from postmenopausal women with or without osteoporosis were detected using RT-qPCR. **(G)** After RAW264.7 cells were transfected with si-NFATc1, the NFATc1 mRNA and protein expression levels were detected using RT-qPCR and western blotting, respectively. **(H)** After the transfection of NFATc1 into RAW264.7 cells, the NFATc1 mRNA and protein expression levels were detected using RT-qPCR and western blotting, respectively. **(I)** After the transfection of RAW264.7 cells with si-YTHDF2, the YTHDF2 mRNA and protein expression levels were detected using RT-qPCR and western blotting, respectively. **(J)** The YTHDF2 mRNA expression levels in human bone samples from postmenopausal women with or without osteoporosis were detected using RT-qPCR. **(K)** After RAW264.7 cells were transfected with si-EIF4A3, the EIF4A3 mRNA and protein expression levels were detected using RT-qPCR and western blotting, respectively. **(L)** The EIF4A3 mRNA expression levels in human bone samples from postmenopausal women with or without osteoporosis were detected using RT-qPCR. **(M)** After RAW264.7 cells were transfected with si-RBM8A, the RBM8A mRNA and protein expression levels were detected using RT-qPCR and western blotting, respectively. **(N)** The RBM8A mRNA expression levels in human bone samples from postmenopausal women with or without osteoporosis were detected using RT-qPCR. These data represent three independent experiments and are presented as the means ± SDs (*p < 0.05).


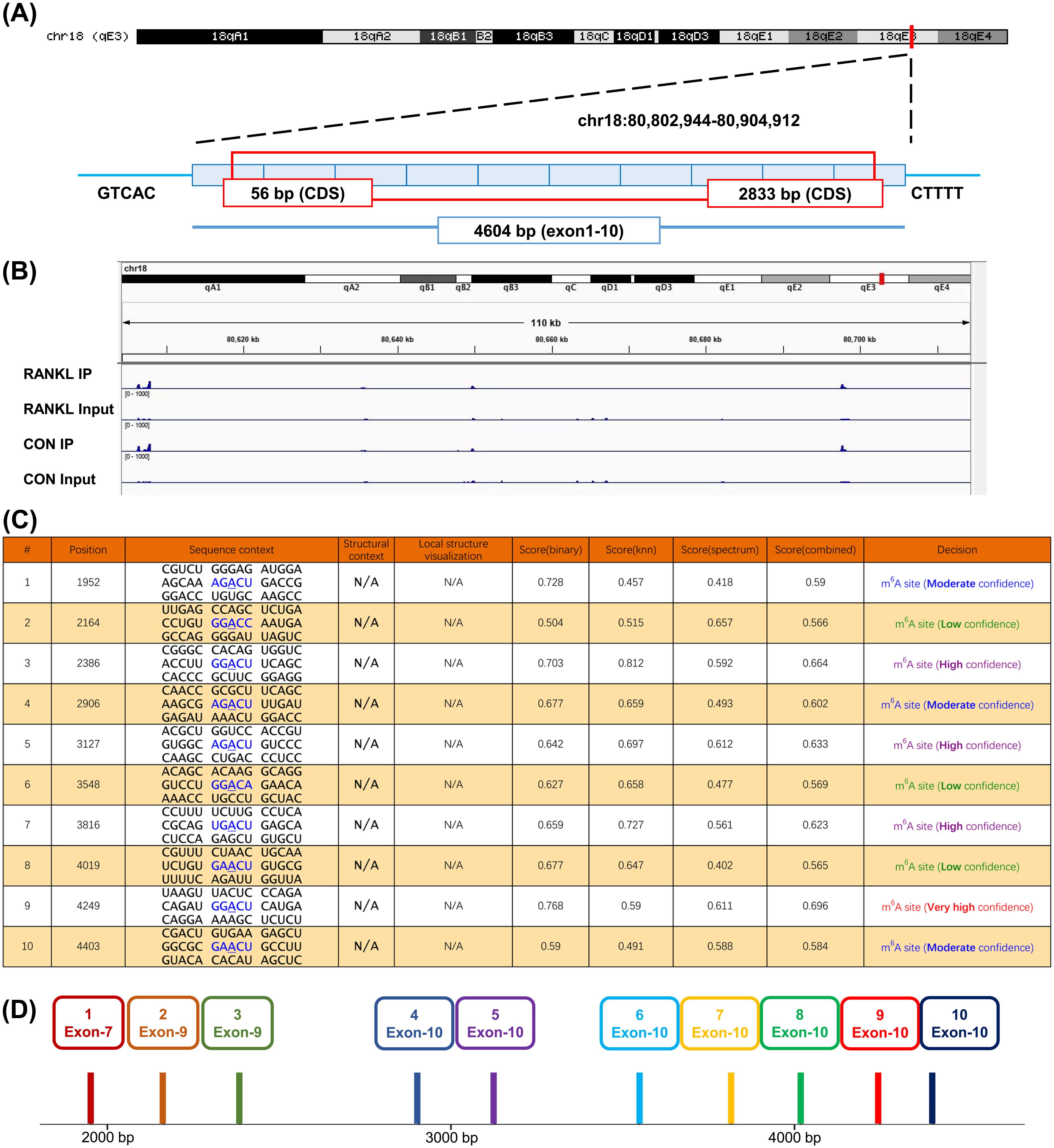


**Fig. S2. (A)** Schematic diagram showing the genomic location of NFATc1. **(B)** Visualization of m6A peaks within the NFATc1 transcript through meRIP-Seq after RANKL stimulation, with m6A peaks located in the 3’ UTR of NFATc1 (IP and Input). **(C)** The potential m6A methylation loci in the NFATc1 gene based on the SRAMP website. **(D)** We divided 10 segments of NFATc1 according to the potential m6A methylation loci.


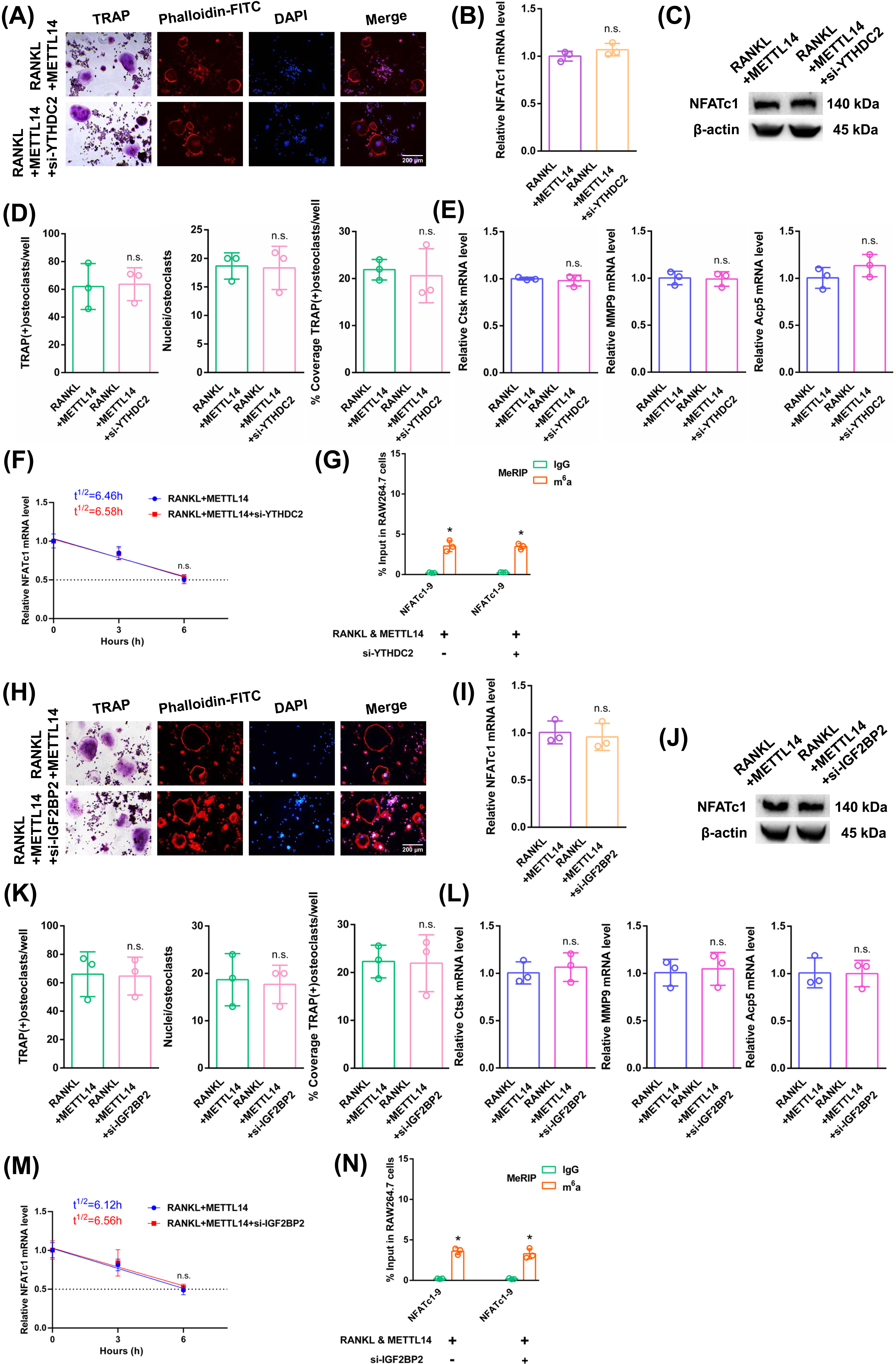


**Fig. S3. (A)** TRAP staining and F-actin band staining were applied to detect osteoclast differentiation after RANKL+METTL14+si-YTHDC2 stimulation. Scale bar: 200 μm. **(B, C)** After RANKL+METTL14+si-YTHDC2 stimulation, the NFATc1 mRNA and protein expression levels in RAW264.7 cells were detected using RT-qPCR and western blotting, respectively. **(D)** Histograms showing the number, coverage rate and nuclei of TRAP-positive osteoclasts after RANKL+METTL14+si-YTHDC2 stimulation. **(E)** Relative expression levels of Ctsk, MMP9 and Acp5 in RAW264.7 cells after RANKL+METTL14+si-YTHDC2 stimulation. **(F)** After RANKL+METTL14+si-YTHDC2 stimulation, the NFATc1 mRNA half-life was estimated via linear regression analysis. **(G)** An m6A-RT-qPCR assay was performed to detect the enrichment of the NFATc1-1 and NFATc1-9 segments after RANKL+METTL14 or si-YTHDC2 stimulation. **(H)** TRAP staining and F-actin band staining were applied to detect osteoclast differentiation after RANKL+METTL14+si-IGF2BP2 stimulation. Scale bar: 200 μm. **(I, J)** After RANKL+METTL14+si-IGF2BP2 stimulation, the NFATc1 mRNA and protein expression levels in RAW264.7 cells were detected using RT-qPCR and western blotting, respectively. **(K)** Histograms showing the number, coverage rate and nuclei of TRAP-positive osteoclasts after RANKL+METTL14+si-IGF2BP2 stimulation. **(L)** Relative expression levels of Ctsk, MMP9 and Acp5 in RAW264.7 cells after RANKL+METTL14+si-IGF2BP2 stimulation. **(M)** After RANKL+METTL14+si-IGF2BP2 stimulation, the NFATc1 mRNA half-life was estimated via linear regression analysis. **(N)** An m6A-RT-qPCR assay was performed to detect the enrichment of the NFATc1-1 and NFATc1-9 segments after RANKL+METTL14 or si-IGF2BP2 stimulation. These data represent three independent experiments and are presented as the means ± SDs (*p < 0.05).


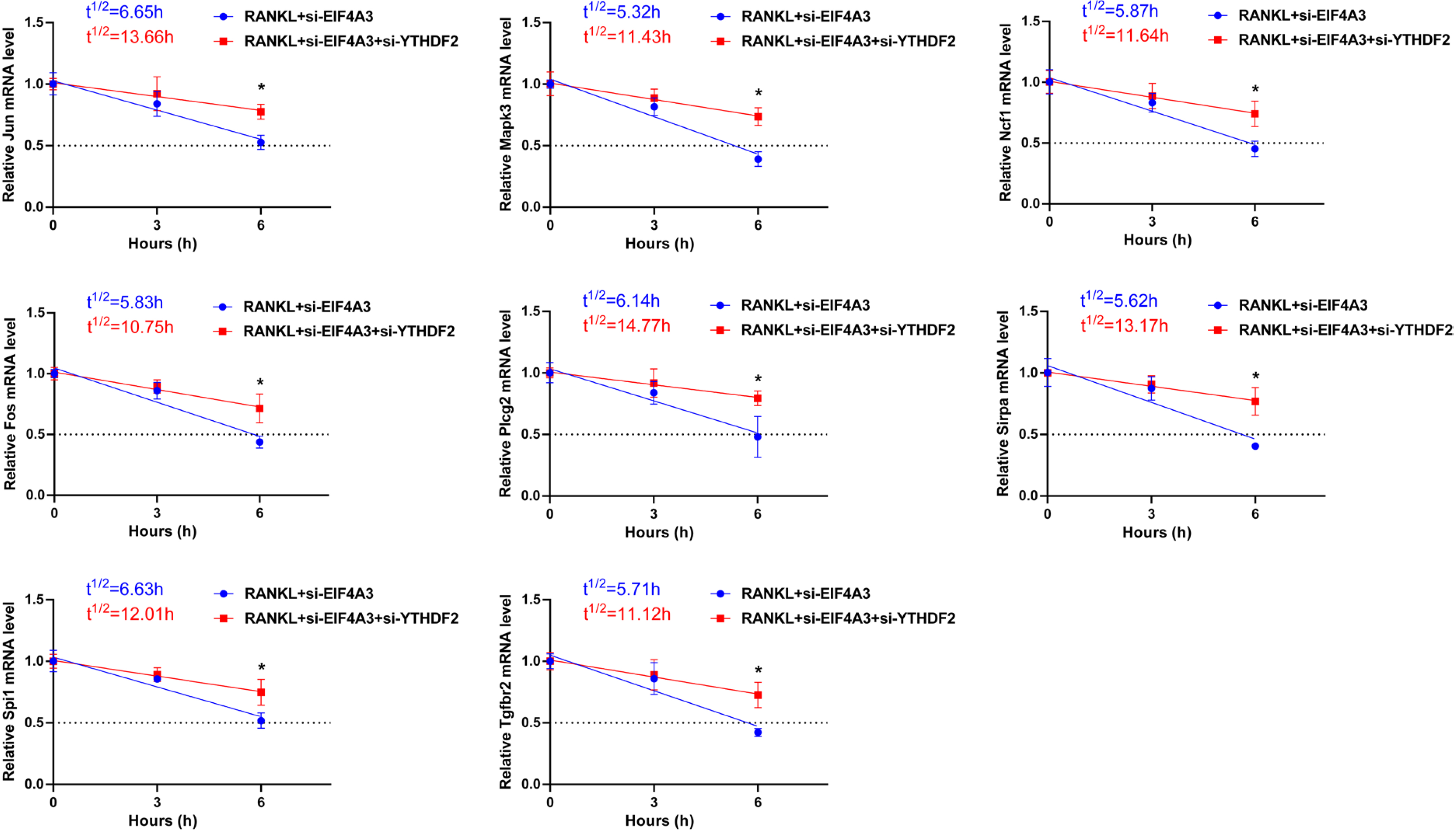


**Fig. S4.** The effects of EIF4A3 and YTHDF2 on the posttranscriptional regulation of osteoclast differentiation-related genes after RANKL+si-EIF4A3+si-YTHDF2 stimulation. After RANKL+si-EIF4A3+si-YTHDF2 stimulation, the half-life of osteoclast differentiation-related mRNAs was estimated via linear regression analysis. These data represent three independent experiments and are presented as the means ± SDs (*p < 0.05).


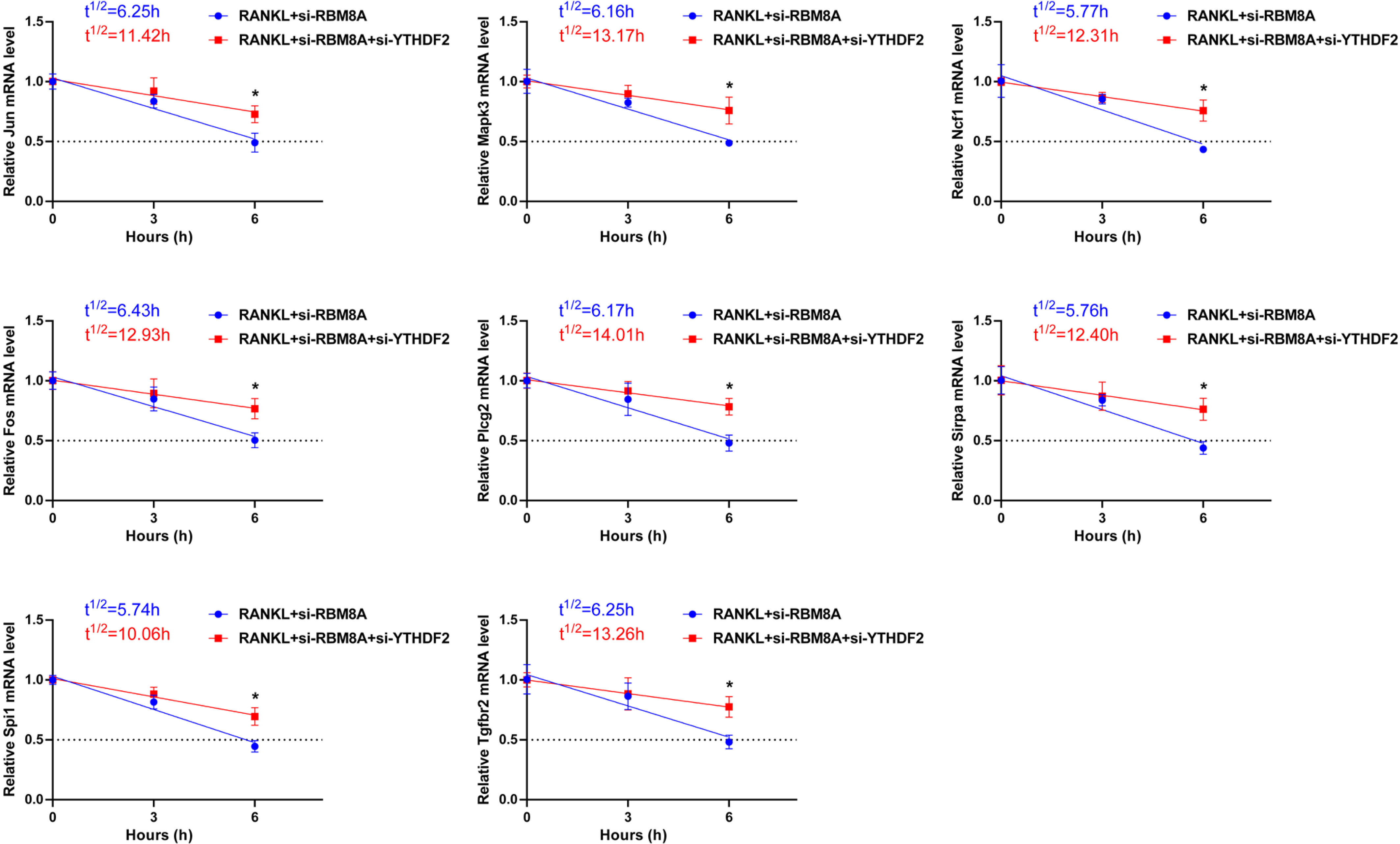


**Fig. S5.** The effects of RBM8A and YTHDF2 on the posttranscriptional regulation of osteoclast differentiation-related genes after RANKL+si-RBM8A+si-YTHDF2 stimulation. After RANKL+si-RBM8A+si-YTHDF2 stimulation, the half-life of osteoclast differentiation-related mRNAs was estimated via linear regression analysis. These data represent three independent experiments and are presented as the means ± SDs (*p < 0.05).


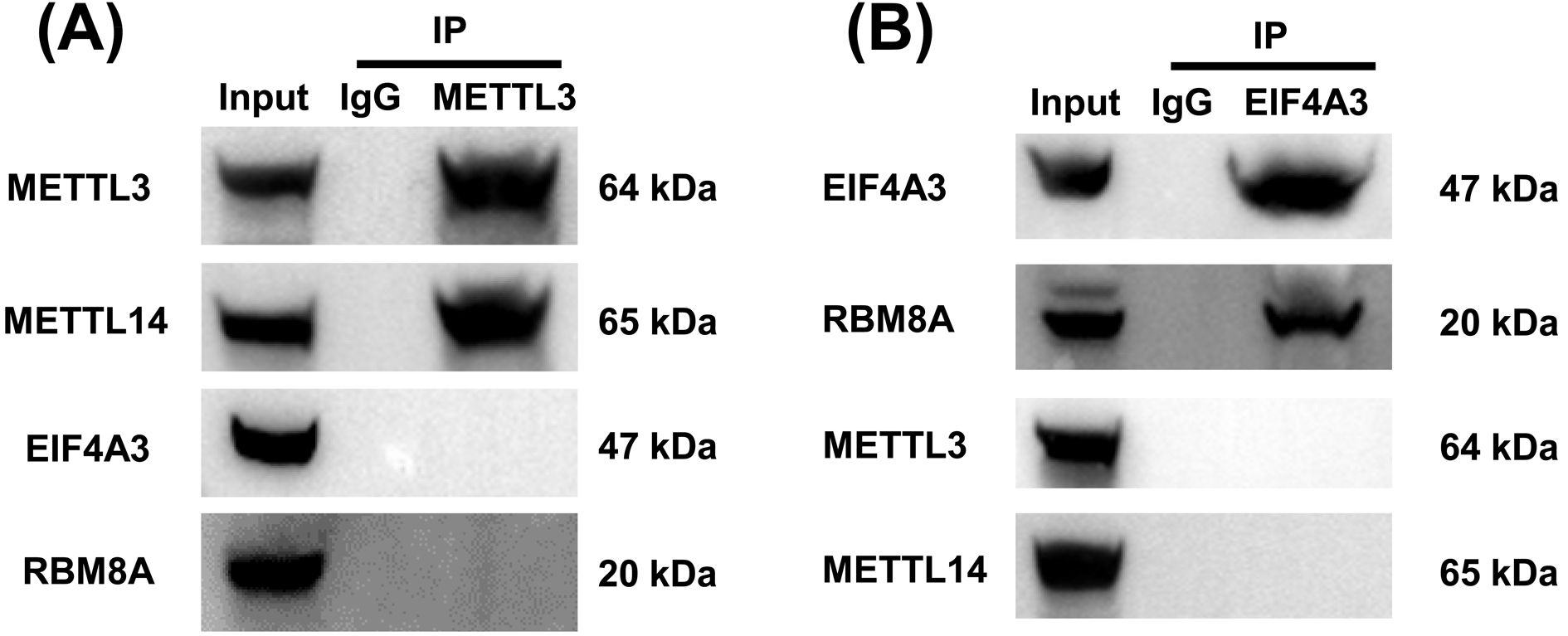


**Fig. S6.** EJCs and METTL3/METTL14 do not coimmunoprecipitate. **(A)** Western blots of EJCs and METTL3/METTL14 proteins from total cell extracts (input) or upon immunoprecipitation with IgG or METTL3. **(B)** Western blots of EJCs and METTL3/METTL14 proteins from total cell extracts (input) or upon immunoprecipitation with IgG or EIF4A3.

**
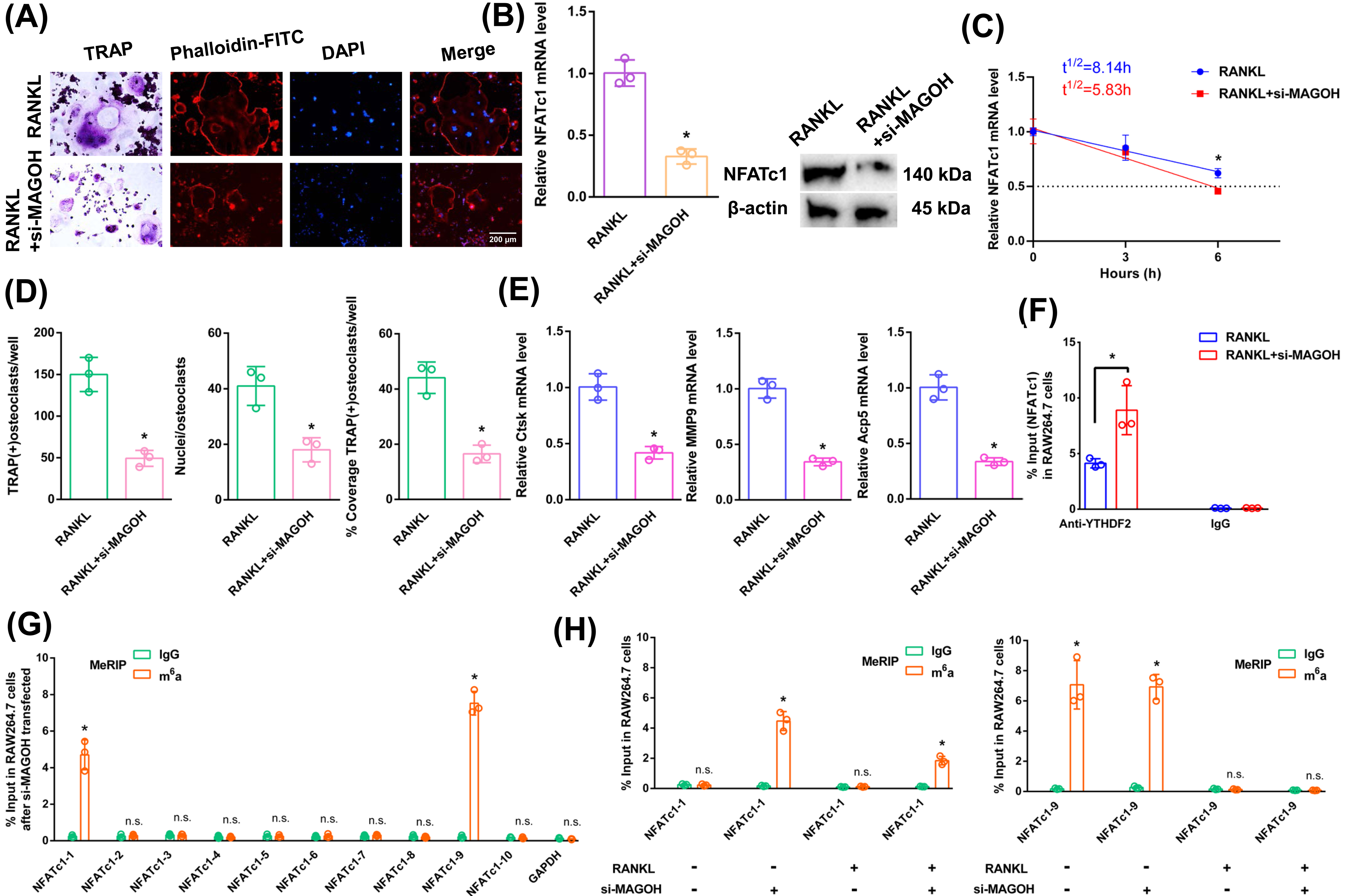
**

**Fig. S7. The effects of MAGOH on the posttranscriptional regulation of NFATc1 expression. (A)** Osteoclast differentiation was assessed by TRAP staining and F-actin band staining after stimulation with RANKL+si-MAGOH. Scale bar: 200 μm. **(B)** The mRNA and protein expression levels of NFATc1 in RAW264.7 cells after stimulation with RANKL+si-MAGOH. **(C)** The half-life of NFATc1 mRNA was estimated by linear regression analysis after stimulation with RANKL+si-MAGOH. **(D)** Histograms display the number, coverage rate and nuclei of TRAP-positive osteoclasts after stimulation with RANKL+si-MAGOH. **(E)** Relative expression levels of Ctsk, MMP9, and Acp5 in RAW264.7 cells after stimulation with RANKL+si-MAGOH. **(F)** Enrichment rate of NFATc1 under RANKL+si-MAGOH condition was detected by RIP assay. **(G)** Enrichment of ten NFATc1 segments between the anti-m6A group and the anti-IgG group after stimulation with si-MAGOH was detected by m6A-RT-qPCR. **(H)** Enrichment of NFATc1-1 and NFATc1-9 segments after stimulation with RANKL or si-MAGOH was detected by m6A-RT-qPCR. These data represent three independent experiments and are presented as the means ± SDs (*p < 0.05).

**Table S1. Primer sequences for RT-qPCR.**

| mmu-Ctsk | F: GGTCCCAGACTCCATCG | R: GCTGAAAGCCCAACAGG |
| --- | --- | --- |
| mmu-MMP9 | F: GACGACATAGACGGCATCC | R: TGGTTCAGTTGTGGTGGTG |
| mmu-Acp5 | F: TTACTACCGTTTGCGCTTC | R: CATTTTGGGCTGCTGACT |
| mmu-NFATc1 | F: TATATGAGCCCATCCTTGCCT | R: GGCTGCCTTCCGTCTCATAG |
| mmu-METTL14 | F: TGGATTTGCATTTTGGCGGG | R: ATGCTATCCGCACTCTCAGC |
| mmu-YTHDF2 | F: CCTGTCGAGCATCACTCCAG | R: TGAGTCACTAGGTCACCTCTCA |
| mmu-EIF4A3 | F: GCAGTTGTCTTTCTGCGGAA | R: ACGCTGCTGAATCGCTGAA |
| mmu-RBM8A | F: CCTTAATTTGGACAGGCGCAC | R: CCAGTCCACACTGATTGGCT |
| mmu-YTHDC2 | F: GCTCATGCAATGATGACCTGT | R: AATGCCATTGTTGAGTCGCC |
| mmu-IGF2BP2 | F: CTACGCCTTCGTGGACTACC | R: TTCAGCCAACAGCCCATC |
| mmu-Jun | F: AGTCCCTTCTCCCGCCTTCC | R: GGTAGCCGCTCGCCTATTTCC |
| mmu-Mapk3 | F: AAGGAGCGGCTGAAGGAGTTG | R: AGGAGCAGGTAGGAGCAGGAC |
| mmu-Ncf1 | F: GCTGGTGGGTGGTCAGGAAAG | R: TCTGTGCGTTGCGGATGGTC |
| mmu-Fos | F: TCCCGTGGTCACCTGTACTCC | R: TGCTGCTGCTGCCCTTTCG |
| mmu-Plcg2 | F: TGGAGCAAGACCGCAGACAAG | R: GGTGCCGTAGAGGATGGTGAAG |
| mmu-Sirpa | F: GAGGACATCCAGCCAGCCAATC | R: GCCACGGAGGGAAGACAAAGG |
| mmu-Spi1 | F: GGGCATCCAGAAGGGCAACC | R: GCCGCTGAACTGGTAGGTGAG |
| mmu-Tgfbr2 | F: ACGACTTGACCTGTTGCCTGTG | R: CCCACCTGCCCGCTGTTG |
| mmu-MAGOH | F: CACCAAAGAAGATGATGCTCTG | R: GGTTGACATCAATAAGGGAACC |
| mmu-GAPDH | F: GTTGCCATCAACGACCCCTT | R: TCCACGACATACTCAGCACC |
| has-NFATc1 | F: GTCCCACCACCGAGCCCACTACG | R: GACCATCTTCTTCCCGCCCACGAC |
| hsa-METTL14 | F: TTTCTCTGGTGTGGTTCTGG | R: AAGTCTTAGTCTTCCCAGGATTG |
| has-YTHDF2 | F: TGTTGGAGAAGCTTCGGTCC | R: ACCCGGCCATGTTTCAGATT |
| has-EIF4A3 | F: TCCAGCAACGAGCAATCAAG | R: GTGGGAGCCAAGATCAAAGC |
| has-RBM8A | F: GAAGCCACCGAAGAAGACAT | R: CCATTGAGTCCCTCCATAGC |
| has-GAPDH | F: AATCCCATCACCATCTTCCAG | R: AAATGAGCCCCAGCCTTC |

**Table S2. The siRNA sequences used in this study.**

| mmu-NFATc1 siRNA | 5’-CCCGUCCAAGUCAGUUUCUAU-3’ |
| --- | --- |
| mmu-METTL14 siRNA | 5’-GGAUGAGUUAAUAGCUAAA-3’ |
| mmu-YTHDF2 siRNA | 5’-GACCAAGAAUGGCAUUGCA-3’ |
| mmu-YTHDC2 siRNA | 5’-CCUGUUAGAUGAUUGCUUUTTT-3’ |
| mmu-IGF2BP2 siRNA | 5’-CCGUUGUCAACGUCACCUAUATT-3’ |
| mmu-EIF4A3 siRNA | 5’-GCUGGACUACGGACAGCACGU-3’ |
| mmu-RBM8A siRNA | 5’-CUGAAUAUGGGGAAAUAAAAA-3’ |
| mmu-MAGOH siRNA | 5’-CCAACAACAGCAAUUACAATT-3’ |

**Table S3. Specific primers for the m6A-modified RNA fragments** **in** **NFATc1.**

| Site 1 | F: ACCACGTCTGGGAGATGGAA | R: TGGTTATCCTCTGGTTGCGGAA |
| --- | --- | --- |
| Site 2 | F: GAAGAGAAGCCAGTACCAGCG | R: TGGCTGTAGTAAGGCCTCGG |
| Site 3 | F: TTCCTCTCCTGCCTACACCAAGG | R: ATGGGTCTCGGCACTTCCT |
| Site 4 | F: CACATCGGAGCTGGCAGAAG | R: GTGGTACCAGATGTGGGTCCAG |
| Site 5 | F: ACACCATCTCAAGGAACGAGAAGG | R: TGCCCTGGTGTGGTCAGACTAAA |
| Site 6 | F: GGAAGTCGAACAGCACAAGGC | R: CATTGCCACCTCTGTCCTTGG |
| Site 7 | F: TTCCATCAGGGTCTACCTCTGGAC | R: AGCTGATGGGCATTTGAGCAC |
| Site 8 | F: CACAGAAGCCTTATAGCTCTCTGC | R: CTAATTACACAGAGCAGACACCAGC |
| Site 9 | F: GAATTGCACCTGTGGCTGGAAG | R: GCCACCATCATACAGACTGACA |
| Site 10 | F: CCCAACGCACAGAAAGAAACA | R: CGGCCAGGAGCTATGTGTGT |

**Table S4. FISH probe.**

| mmu-NFATc1 | GAATTGCACCTGTGGCTGGAAG |
| --- | --- |

**Table S5. Specific primers for the conditional knockout mice.**

| EIF4A3 | F: TCACAGGTAATCTCGTAGGCTGG | R: AAGCTAATGAAAAAGTATCTTGG |
| --- | --- | --- |
| RBM8A | F: CCCCCTTGGGAAAGGAATTAGGG | R: TAAGGAAAAGAGTACTTGGTTGG |

**Table S6. Mus musculus nuclear factor of activated T cells, cytoplasmic, calcineurin dependent 1 (Nfatc1), transcript variant 5, mRNA.**

| Nfatc1 |
| --- |
| GTCACGCCGGGGACGCGCGCGAGCGCCGGGCGCACCAGTGCCGCGCGACCCCGACATGACGGGGCTGGAGCAGGACCCGGAGTTCGACTTCGATTTCCTCTTCGAGTTCGATCAGAGCGGCGGGGGCGCCGCGGCCGCAGAACACTACAGTTATGTGTCCCCTAGTGTCACCTCGACCCTGCCCCTTCCCACAGCACACTCTGCCTTGCCAGCAGCATGCCACGACCTCCAGACGTCCACCCCGGGTATCTCAGCTGTTCCTTCAGCCAATCATCCCCCCAGTTACGGAGGGGCTGTGGACAGCGGGCCTTCGGGATACTTCCTGTCCTCTGGCAACACCAGACCCAACGGGGCCCCGACTCTGGAGAGTCCGAGAATCGAGATCACCTCCTACCTGGGCCTACACCATGGCAGCGGCCAGTTTTTCCACGACGTGGAGGTGGAAGACGTACTTCCTAGCTGCAAGCGCTCACCGTCTACAGCAACCCTGCACCTGCCCAGCCTGGAAGCCTACAGAGACCCCTCCTGCCTGAGCCCAGCCAGCAGTCTCTCCTCCAGAAGCTGTAACTCTGAGGCCTCCTCCTACGAGTCCAACTACTCCTACCCATACGCGTCCCCCCAGACCTCTCCGTGGCAGTCACCCTGCGTGTCTCCCAAGACCACGGACCCGGAGGAGGGTTTTCCCCGAAGCCTGGGTGCCTGCCACCTGCTAGGATCGCCCAGGCACTCCCCATCCACCTCTCCTCGGGCAAGCATCACGGAGGAGAGCTGGCTCGGTGCCCGCGGCTCCCGGCCCACGTCCCCCTGCAACAAGCGCAAGTACAGTCTCAATGGCCGGCAGCCCTCCTGCTCACCCCACCACTCACCCACACCATCCCCCCATGGCTCCCCTCGGGTCAGTGTGACCGAAGATACCTGGCTCGGTAACACCACCCAGTATACCAGCTCTGCCATTGTGGCAGCCATCAACGCCCTGACCACCGATAGCACTCTGGACCTGGGTGATGGGGTCCCTATCAAGTCTCGAAAGACAGCACTGGAGCATGCGCCCTCTGTGGCCCTCAAAGTAGAGCCAGCTGGGGAAGACCTGGGCACCACTCCACCCACTTCTGACTTCCCACCCGAGGAGTACACCTTCCAGCACCTTCGGAAGGGTGCCTTTTGCGAGCAGTATCTGTCGGTGCCACAGGCCTCGTATCAGTGGGCGAAGCCCAAGTCTCTTTCCCCGACATCATATATGAGCCCATCCTTGCCTGCCCTTGACTGGCAGCTCCCGTCACATTCTGGTCCATACGAGCTTCGGATCGAGGTGCAGCCCAAGTCTCACCACAGGGCTCACTATGAGACGGAAGGCAGCCGGGGGGCTGTGAAGGCTTCAGCTGGAGGACACCCCATTGTGCAGCTACACGGTTACTTGGAGAATGAACCTCTCACGCTACAGCTGTTCATTGGGACGGCTGACGACCGCCTGCTGAGGCCCCACGCCTTCTACCAGGTCCACCGGATCACGGGGAAGACTGTCTCCACCACCAGCCACGAGATCATCCTGTCCAACACCAAAGTCCTGGAGATCCCGTTGCTTCCAGAAAATAACATGCGAGCCATCATCGACTGTGCTGGGATCCTGAAGCTCAGAAACTCTGATATTGAGCTGAGGAAAGGGGAGACAGACATCGGGAGGAAGAACACCAGGGTGAGGCTGGTCTTCCGAGTTCACATCCCACAGCCCAATGGCCGGACGCTGTCTCTCCAGGTGGCCTCGAACCCTATCGAGTGTTCCCAGCGGTCAGCCCAGGAGCTGCCCCTCGTGGAGAAGCAGAGCACAGACAGCTACCCAGTCATCGGCGGGAAGAAGATGGTGCTGTCTGGCCATAACTTTCTGCAAGACTCCAAAGTCATTTTCGTGGAGAAGGCTCCAGATGGCCACCACGTCTGGGAGATGGAAGCAAAGACTGACCGGGACCTGTGCAAGCCAAATTCCCTGGTGGTTGAGATACCACCTTTCCGCAACCAGAGGATAACCAGCCCCGTCCAAGTCAGTTTCTATGTCTGCAACGGGAAACGGAAGAGAAGCCAGTACCAGCGTTTCACGTACCTTCCTGCCAATGTTCCAATTATAAAGACAGAACCCACGGACGACTTTGAGCCAGCTCTGACCTGTGGACCAATGAGCCAGGGGATTAGTCCTCTGCCGAGGCCTTACTACAGCCAACAGCTCACCATGCCTCCCGACCCCGGCTCCTGCCTCGTGGCTGGCTTCGCCCCCTGCTCCCAGAGGAACACGCTGATGCCCACGCCTCCCAACGCAAGCCCGAAGCTCCACGACCTTTCCTCTCCTGCCTACACCAAGGGCCTCACCAACCCGGGCCACAGTGGTCACCTTGGACTTCAGCCACCCGCTTCGGAGGCCCCCACCATGCAGGAAGTGCCGAGACCCATGGCCATCCAACCCAACTCGCCTGAGCAGCCCCCATCCGCCAGGCTACAGCCGCAGGTGAGTCCACATCTGAACAGTAGCTGTCCCCTTGGTCGCCGACAAGTGCTCTGTCCCAACAGCCCCTCTTCTCCACTTCCATCTGCTGCCCAAGAGCCAGCCTGCTTACAGTCCTCAGCCCTCCCTCCTGACATGGGCCACCGGCAGCCACAACCGCAGAAGGTTCAAAGAAATGAATCTCCAGCCGTATTGCCAGAGGTGTGTGAGGACAGTGGCCATAACTTGGCCCCTATTCCTGTAGTGATCAAGCAAGAGCCTGAGGAATTGGACCAGTTGTACTTGGATGATGTAAATGAGATCATACGTAACGACCTCTCCAGCACGATCCCCCACTCCTAATTCAGCACATCGGAGCTGGCAGAAGCTGTGAGGAGTTGGCTCAGTGCAGACAACCGCGCTTCAGCAAGCGAGACTTTGATGAGATAAACTGGACCCACATCTGGTACCACTCAGAAATCCCAACTTACCGAACGAACGGAACGCTAGGAGCTTATGTCTGAAGAAGTAGCTCTCCAACAGGGACGGAGGAGCAGGAGAGGGCCCACTGCATCTCCTGAAAGAAACACCATCTCAAGGAACGAGAAGGGCTGGTCAGTGAGGCCAGGGGCAGACGCTGGTCCACCGTGTGGCAGACTGTCCCCAAGCCTGACCCTCCTGCCCACTGGATCAAAACACTGGAAGTGCCTTATTTAGTCTGACCACACCAGGGCATATGGAAGTGAGCACTGAATTTTCTACCATGAGAGTATTTGTGGGAGCCAAAGCTATTAAAGAACACTTCCTTAGGAGGTTTTATCTTTTGTATTAATTGATAGGATTCTGAAGAGCCTCCTGCGTATCTCTAGCTTTTCTGGCTGCCCTACACAATTTGCCTGCCTTATCCAGTGCATTTTAGAGGTCTTCCAGCCTGTCTTCTTGGCTCTCTCATAGTCAGCCCCCTCCTCTGTGTGACTGAAACCACAGGTCTGTTAATAATAGCATGCTAGGATTCTTGTTCTCAATCCAGCTTCGAACATAGTGAAGGAAGTCGAACAGCACAAGGCAGGGTCCTGGACAGAACAAAACCTGCCTGCTACAGGACAGAGCTTGTGTTTGCATGCATGTACATATAGGCATATATTTATGTATAGATATCCAAGGACAGAGGTGGCAATGAGCAGTCTGTGCCCACCAGGGGCCTTCCTTCCATGTTAGCAATAACCAGTATCCACCTTGAGAGTGCACCTCAGAGTCCAGAACCGGCTTCCCACCATCATGGTCTTTCCTTCCATCAGGGTCTACCTCTGGACAGGCAGGTGGCCTTTTCTTGCCTCACGCAGTGACTGAGCACTCCAGAGCTGTGCTCAAATGCCCATCAGCTACCAAATGGCAAAATCTGAAAGTGGTTGTAAATAACCATTACAGAATGAGTGTAGTATATTTGTTCAATTATAAGATTATTCTTTCACAGAAGCCTTATAGCTCTCTGCTTCATCTAAGAAAACAATTACCAAAAAAAACAACGTTTCTAACTGCAATCTGTGAACTGTGCGTTTTCAGATTGGTTACTGGTAACAGATAAGCTGGTGTCTGCTCTGTGTAATTAGCTGCTTACTTCAGTTACTAGCAGTGACCTATTATTTCTTATAACCAAAAAAAGCATGGTTTAATTAAAACATGTTTAATGATCGTGCCTTAGGAGTTAATGCCCCCTTATGGAACACGCCTGAATTGCACCTGTGGCTGGAAGTTTTAAGTTACTCCCAGACAGATGGACTCATGACAGGAAAAGCTCTCTCACAGGAAGATGCATCTTTAAAATTTTTGTCAGTCTGTATGATGGTGGCTTACCTTTCCCAACGCACAGAAAGAAACAACTGTCTGAAAGCATACTGAATGATTTCGCACGACTGTGAAGAGCTGGCGCGAACTGCCTTGTACACACATAGCTCCTGGCCGCCTGCAGGCTGCCTCCCGCCTGCCTCTCGTCTGTACCCCATGTTTATTAGCATCATGGAGTTGCATGAACCATTCTTAGTAGACTGTCATCTGAAAGCAAGCGTTTGATATTTGTGTCAGCTATCTTTGTAGTTAGGAGATGAATCCAATAAAGCAGTATTTTTTTTCTTTT |
| Blue words indicated the region of NFATc1 gene 5' or 3' untranslated regions. Red words indicated the m6A methylation sites of NFATc1 gene. |
